# Bioactive Compounds from Kefir and Their Potential Benefits on Health: A Systematic Review and Meta-Analysis

**DOI:** 10.1155/2021/9081738

**Published:** 2021-10-27

**Authors:** Carla P. Vieira, Anisio Iuri L. S. Rosario, Carini A. Lelis, Bruna Samara S. Rekowsky, Anna Paula A. Carvalho, Denes Kaic A. Rosário, Thaísa A. Elias, Marion P. Costa, Debora Foguel, Carlos A. Conte-Junior

**Affiliations:** ^1^Center for Food Analysis (NAL), Technological Development Support Laboratory (LADETEC), Federal University of Rio de Janeiro (UFRJ), Cidade Universitária, Rio de Janeiro, RJ 21941-598, Brazil; ^2^Laboratory of Advanced Analysis in Biochemistry and Molecular Biology (LAABBM), Department of Biochemistry, Federal University of Rio de Janeiro (UFRJ), Cidade Universitária, Rio de Janeiro, RJ 21941-909, Brazil; ^3^Laboratory of Inspection and Technology of Milk and Derivatives, Escola de Medicina Veterinária e Zootecnia, Universidade Federal da Bahia, 40170-110 Bahia, Brazil; ^4^Laboratory of Protein Aggregation and Amyloidosis, Instituto de Bioquímica Médica, Universidade Federal do Rio de Janeiro, 21941-590 Rio de Janeiro, Brazil; ^5^Graduate Program in Sanitary Surveillance (PPGVS), National Institute of Health Quality Control (INCQS), Oswaldo Cruz Foundation (FIOCRUZ), Rio de Janeiro, RJ 21040-900, Brazil

## Abstract

Despite evidence of health benefits from kefir administration, a systematic review with meta-analysis on bioactive compounds associated with these benefits is still absent in the literature. Kefir is fermented milk resulting from the metabolism of a complex microbiota in symbiosis. Recent researches have investigated the bioactive compounds responsible for the preventive and therapeutic effects attributed to kefir. However, differences in functional potential between industrial and artisanal kefir are still controversial. Firstly, we identified differences in the microbial composition among both types of kefir. Available evidence concerning the action of different bioactive compounds from kefir on health, both from *in vitro* and *in vivo* studies, was subsequently summarized to draw a primary conclusion of the dose and the intervention time for effect, the producer microorganisms, the precursor in the milk, and the action mechanism. Meta-analysis was performed to investigate the statistically significant differences (*P* < 0.05) between intervention and control and between both types of kefir for each health effect studied. In summary, the bioactive compounds more commonly reported were exopolysaccharides, including kefiran, bioactive peptides, and organic acids, especially lactic acid. Kefir bioactive compounds presented antimicrobial, anticancer, and immune-modulatory activities corroborated by the meta-analysis. However, clinical evidence is urgently needed to strengthen the practical applicability of these bioactive compounds. The mechanisms of their action were diverse, indicating that they can act by different signaling pathways. Still, industrial and artisanal kefir may differ regarding functional potential—OR of 8.56 (95% CI: 2.27–32.21, *P* ≤ .001)—according to the observed health effect, which can be associated with differences in the microbial composition between both types of kefir.

## 1. Introduction

Fermentation of a matrix produces kefir. Milk is the matrix generally used, resulting in a beverage acidic, slightly alcoholic, and with a creamy consistency [1]. It results from milk fermentation by microorganisms that live in symbiosis in kefir grains. Kefir differs from other fermented milk because it is a metabolic result of a diversity of microorganisms. Lactose fermenting and nonfermenting yeast species (*Kluyveromyces*, *Pichia*, and *Saccharomyces*), with a predominance of lactic acid bacteria (*Lactobacillus*, *Lactococcus*, *Leuconostoc*, and *Streptococcus*), besides acetic acid bacteria [2] make up the grain's microbiota.

In recent years, there has been an increase in scientific research on kefir motivated by the association of beverage consumption with therapeutic effects [3]. Regular consumption of kefir has been associated to the reduction of severity of inflammatory bowel disease [4], antihypertensive effect [5], anticarcinogenic effect [6], increased insulin sensitivity [7], improved lipid profile [8], therapeutic effects on osteoporosis [9], and neurodegenerative disease [10]. The positive health effects have been related to the antioxidant capacity [11] and modulation of the intestinal microbiota [12] by the kefir drink. Bioactive compounds present in kefir, produced by microorganisms during fermentation and storage of beverage, have been attributed to these benefits; kefiran, exopolysaccharides, bioactive peptides, and organic acids are the bioactive compounds commonly implicated with the therapeutic potential of kefir [13–17]. However, there is still a need for a deeper discussion about the bioactive compounds present in the kefir drink [13] to distinguish them according to their therapeutic potential for each disease.

In addition, the possible difference in the functional potential between artisanal and industrial kefir is controversial in the literature. The use of kefir grains results in artisanal kefir [18], while previously selected starter culture of bacteria and yeast species leads to commercial or industrial kefir [19]. Some studies have reported artisanal kefir with greater therapeutic potential due to its greater microbiological diversity [20, 21]. In contrast, other studies have described industrial kefir as promising in treating diseases [22], while Ebner et al. [23] have found no significant difference between both. In this context, a meta-analysis could be helpful to elucidate the inconsistencies observed between studies.

Thus, there have been some reports on bioactive compounds from kefir and health benefits in recent years. However, there is still a lack of an overview and in-depth approach in this research field, so a systematic review with meta-analysis will be relevant for this purpose. Therefore, it is necessary to summarize the bioactive compounds from kefir produced by different microorganisms that make up its microbiota, their beneficial effects, action mechanisms, and their precursors in the milk. Based on this knowledge, it will be possible to provide a theoretical basis for developing functional formulations by the food industry to prevent specific diseases. In addition, the pharmaceutical industry may prepare formulations with therapeutic potential from bioactive compounds isolated from kefir. In this scenario, this review summarized the antimicrobial, antioxidant, immune-modulatory, gut microbiota-modulatory, anticancer, antiosteoporosis, antihypertensive, antidiabetic, and lipid profile-modulatory role of the bioactive compounds from milk's kefir. Prevention and treatment of neurodegenerative diseases also were covered. The functional differences between both types of kefir finished this review.

## 2. Methods

### 2.1. Focus Questions

The development of the question was according to the population, intervention, comparison, and outcome (PICO) method. The questions formulated were as follows: What are the bioactive compounds in milk kefir and their producing microorganisms? What are the precursors of these bioactive compounds in milk? What are the mechanisms of action of the main bioactive compounds in kefir? Do bioactive compounds differ in terms of concentration and intervention time to obtain the same effect on health? Is artisanal kefir drink more functional than the one produced industrially?

### 2.2. Data Collection Process and Eligibility Criteria

Two authors (C.P.V and A.P.A.C) independently conducted the preliminary selection of identified abstracts and titles of research articles published in English; we removed the time filter not to limit the number of manuscript resulting. Thus, the initial screening publications covered the period from 1986 to 2021. Abstracts were then removed in this initial screening if the papers did not investigate any of the following health aspects: antimicrobial activity, antioxidant activity, effect on cancer, neurodegenerative diseases, lipid profile, blood pressure, plasma glucose, gut microbiota modulation, inflammation, and osteoporosis both *in vitro*, in situ, *in vivo* animal, and human clinical trials. The criterion used to choose these specific health benefits was that they had been the most investigated in the scientific literature in the last two decades [24–26]. Papers about nonmilk kefir were excluded, including editorials, letters, reviews, commentaries, monographs, preprints, and Ph.D. thesis. Based on the entire reading of the paper, all studies included in the present work were controlled experiments and with a quantitative approach for data analysis. Only studies in which it was possible to determine the bioactive compounds responsible for the observed health effect were included. Studies addressing the microbial composition of kefir grains or starter culture of milk kefir were also included. Some studies considered essential to compose the present work such as those that address conventional therapies, recent reviews on the effect of kefir, and pathology of the diseases studied that was not included in any of the research bases were added to compose our introduction and discussion.

Finally, we summarized information about the type of kefir (artisanal or industrial) from which the bioactive compound was derived, the study model, the definition of the bioactive compound, and its effects compared to the control treatment. The results were reported in agreement with the Preferred Reporting Items for Systematic Review and Meta-Analyses (PRISMA) statement.

### 2.3. Information Sources

Our search protocol strategy used search strings constructed and adapted for five electronic databases: Science Direct, Pubmed, Embase, Web of Science, and Scopus. The initial screening process was conducted from February 2021 to March 2021. Also, directed searching was carried out by checking the reference lists of relevant articles. The research questions were used to summarize the search strings through which the manuscripts were recovered. The string was based on predetermined groups of keywords related to microbial composition of artisanal and industrial milk kefir, bioactive compounds in the milk kefir, and their effect on health, as follows:

- *Search Component 1*. microorganisms OR “microbial composition” OR “kefir grains” OR “starter culture” AND “milk kefir”
- *Search Component 2*. “bioactive compounds” OR “functional compounds” AND “milk kefir”
- *Search Component 3*. “health benefits” OR “health effects” OR “functional effects” AND “milk kefir”

### 2.4. Risk of Bias Assessment

Possible sources of bias include eligibility criteria, the impact of missing data, missing primary results, chosen database, chosen language (English), and article type selected for our review.

### 2.5. Meta-Analysis

Two authors (A.I.L.S.R and D.K.A.R) to conduct the meta-analysis extracted data from the included articles. Any inconsistency in retrieved data was solved by discussion. *In vitro*, *in situ*, and *in vivo* papers were pooled for examination. From every publication, each different outcome point was extracted as an independent study. Then, we investigated a statistically significant difference between intervention and control. However, only health effects with an appropriate number of studies were included in the meta-analysis: antimicrobial (including eight groups of microorganisms, totalizing 182 *in vitro* and *in situ* studies from 15 papers), antioxidant (149 *in vitro* and *in situ* studies included in 7 articles), anticancer (44 *in vitro* studies comprising five publications), immune modulation (271 *in vitro* and *in vivo* studies from 11 papers), and microbiota modulation (40 *in vitro* and *in vivo* studies retrieved from 5 publications) effects.

The analyses were conducted evaluating the presence or absence of health benefits for the different outcomes. The definitions of presence/lack of action varied across publications, as different methodologies were used on the selected studies. In this case, the resolution was directed by three authors based on the specific outcome. For example, considering antimicrobial research, if a result was expressed in log UFC, decreasing log UFC by kefir treatment was deemed “presence of action.” Similarly, if a study result was defined as an inhibition zone, reducing zone size (mm) by kefir treatment was considered a “lack of action”.

In addition, to measure the heterogeneity among studies, *I*^2^ test was used. The *I*^2^ assumes the null hypothesis that all the studies are homogeneous or that each study is measuring an identical effect so that a *P* value tests this hypothesis. In this scenario, the *I*^2^ statistic describes the percentage of variation across studies due to inconsistency (heterogeneity) rather than sampling error (chance). A significance level of 0.05 was used herein. Studies with *I*^2^ ≤ 50% were considered homogenous. The *I*^2^ was quantified as follows [27]:

(1) $$\begin{array}{c} I^{2}\;\left(\%\right)=\left(\frac{Q-d_{f}}{Q}\right).100, \end{array}$$

where *I*^2^ is the inconsistency across studies, *Q* is the Cochran's heterogeneity statistic, and *d*_*f*_ is the degrees of freedom.

Subsequently, functional differences between artisanal and industrial kefir were investigated for health effects. Both types of kefir were tested: antimicrobial (182 *in vitro* and *in situ* studies) and antioxidant activity (149 *in vitro* and *in situ* studies). Then, studies were analyzed to compare the health effects of consuming artisanal kefir drinks rather than the industrial variety, evaluated by odds ratio (OR) and corresponding 95% confidence intervals (CIs). All analyses were performed using Review Manager 5.4 (Cochrane Collaboration, London, UK).

## 3. Results

### 3.1. Literature Search

**Figure 1:**
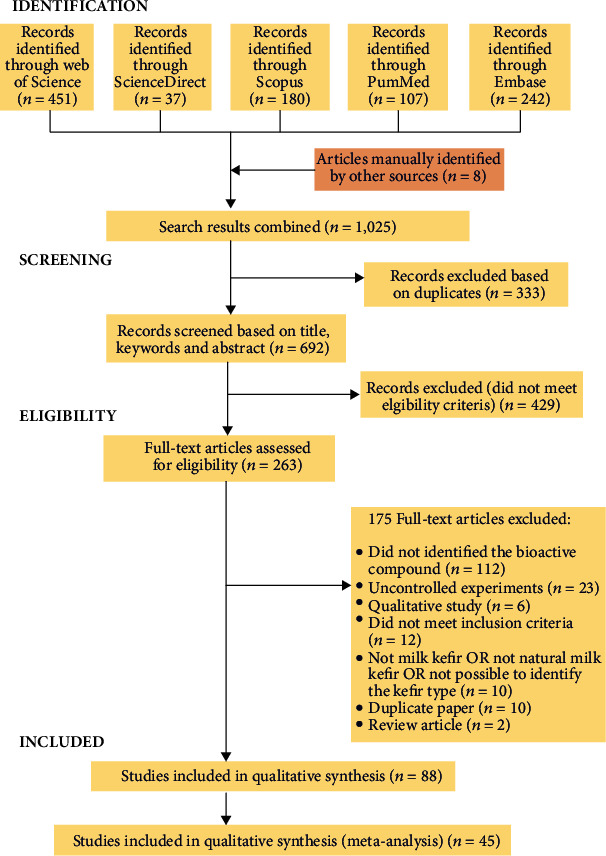
PRISMA flow diagram with results of the systematic search.

There were 451 articles identified at Web of Science, 242 at Embase, 180 at Scopus, 107 at Pubmed, and 37 at Science Direct. Still, 8 articles were manually identified by other sources for addition of effect on neurodegenerative disease. Of these, 333 were duplicates or triplicates and were excluded, with 692 papers remaining. After reading the titles and abstracts, 263 papers were selected for the full read, but only 88 met the eligibility criteria (Figure 1). Among the articles read in full, the main reasons for exclusion were as follows: nonidentification of the bioactive compound (*n* = 112) and uncontrolled experiments (*n* = 23). This highlights the need for experiments with a more elaborate experimental design, as well as that identify the bioactive compound responsible for the health effects observed due to the use of kefir.

### 3.2. Meta-Analysis: Study Selection and Characteristics

Data extraction for meta-analysis consisted of 45 papers. Studies were conducted in Argentina, Brazil, Canada, China, Egypt, Iran, Italy, Japan, Malaysia, South Korea, Thailand, Taiwan, Turkey, the United Kingdom, and the United States of America. The publication year of studies ranged from 2005 to 2021. The concentration of kefir used in the studies varied between 0.01 and 1000 mg/mL. The interventions lasted between 0.5 and 1344 hours.

### 3.3. Structure and Microorganisms of the Kefir Grains

Kefir is a beverage commonly produced from milk and involves a complex fermentative process from the microbiological diversity in “kefir grains” [28]. Typically, kefir grains are inoculated into milk in proportions of 5% to 10% [29], which gives characteristics such as a creamy fermented milk with a slightly acidic taste depending on the starter culture and mixture of the inoculated microorganisms [1].

The diversity of the “kefir grains” embraces numerous symbiotic interactions between lactic acid bacteria (LAB), acetic acid bacteria, mycelial fungi, and yeasts [2], resulting in an acidic and alcoholic fermentation [30]. These microorganisms present in kefir are interspersed in a matrix composed of proteins and polysaccharides [31]. Macroscopically, this matrix can be characterized as solid, cauliflower-like grains viscous and firm consistency, with a color that varies from white to yellowish [32, 33].

According to Khokhlacheva et al. [34], the consortium between microorganisms that evolves an adaptive capacity and enzymatic activity is the key mechanism for their development and survival in milk kefir. In kefir grains, the lactic acid bacteria (LAB) usually present greater levels when compared with acetic acid bacteria. This composition presents itself dynamically and generally changes according to the fermentation time, where the dominance of *Lactobacillus kefiranofaciens* can be observed in the early stages of fermentation, giving rise to the most prominent growth of *Leuconostoc mesenteroides* in the final stages of the process [35]. Several studies around the world are aimed at determining the composition of kefir microbiota (Table 1) since it varies according to geographic, climatic, and cultural factors [33, 36].

The inoculated kefir grains ferment the artisanal kefir [18], while a previously selected starter culture of bacteria and yeast species results in commercial or industrial kefir [19]. According to Table 1, although the *Lactobacillus* and *Lactococcus* genera predominate in both types of kefir, *Enterococcus* was not reported in industrial kefir. Another important aspect can be pointed out in industrial kefir by commercial species not commonly found in artisanal kefir: *Saccharomyces boulardii*, *Lactobacillus lactis*, *Bifidobacterium lactis*, *Bifidobacterium longum*, *Bifidobacterium breve*, and *Lactobacillus reuteri*. Korsak et al. [29] corroborated these findings, reporting that industrial kefir is typically composed of selected cultures and conventionally used in dairy products.

Concerning fungal composition, although both types of kefir have presented common species, such as *Saccharomyces cerevisiae*, *Kluyveromyces marxianus*, and *Kazachstania unispora*, the fungal diversity in artisanal kefir was dramatically greater. In addition, *Kazachstania exígua*, *Kazachstania turicensis*, and *Saccharomyces florentinus* were reported only in industrial kefir (Table 1). Other differences between both kefirs include differences in efficiency under conditions of nutrient competition [41], location and adhesion of microorganisms in the structure of kefir grains, or even different abilities of species to grow in milk [18].

However, more microbiota characterization from artisanal and industrial kefir is necessary to define the microbial similarities and particularities between these two types of milk kefir. This characterization is highly relevant since the beverage's bioactive compound profile is closely related to the producing microorganisms present [42, 43]. The use of kefir grains in the artisanal beverage is correlated to higher counts in the final product and remarkable survival during passage through the gastrointestinal tract, proving significant probiotic properties compared to industrial cultures [42]. Thus, the analysis proposed here may justify possible functional differences between both types of kefir.

Kefir grain's microorganisms can present the ability to produce bioactive compounds during the fermentation and storage of kefir beverages. Consistently, from 48 strains isolated from Russian kefir grains, ten species of *Lactobacillus* sp. were recognized with probiotic potential [39]. Some yeast strains, such as *Saccharomyces cerevisiae* KU200284, present double importance: a starter culture and a probiotic [44]. In Korean kefir, the acetic acid bacterial strain *Acetobacter fabarum* DH1801 had viability as a functional starter with food preservative mechanisms and the potential as a probiotic agent [45]. In addition, the species of LAB has a fundamental role in the formation of exopolysaccharide (EPS), which is a significant bioactive compound in kefir [37]. In this scenario, the *Lactobacillus kefiranofaciens* is considered the main piece in the formation of kefir grains [18] since its genes demonstrate a great capacity to produce exopolysaccharides (such as kefiran) which make up the structure of the kefir grain [43]. Similarly, *Lactococcus lactis* ssp. *cremoris* MRS 47 does found for Vieira et al. [38] to be capable of producing conjugated linoleic acid (CLA), a bioactive compound, from milk fat.

### 3.4. Bioactive Compounds from Kefir and Their Effects on Health

#### 3.4.1. Antimicrobial Activity

The antimicrobial activity of kefir was mainly attributed to exopolysaccharides (EPSs), specially kefiran, and organic acids, especially lactic acid. However, bioactive peptides with antimicrobial activity have also been identified (Table 2).

Regarding the producing microorganisms, kefiran and EPS were produced by *Lactobacillus kefiranofaciens*, while bioactive peptides were synthesized by *Lactobacillus paracasei*, species belonging to the *Lactococcus* genus and yeast. Biofilms and S-layer proteins, turn on, were produced by *Lactobacillus plantarum* and *Lactobacillus kefir*, respectively (Table 2). It demonstrates that microorganisms of the *Lactobacillus* genus are relevant for the production of bioactive compounds with antimicrobial activity in kefir. In addition, among the 18 articles selected from the systematic review addressing the antimicrobial effect of the compounds, only 1 of them investigated the precursor of the compound in milk; thus, the investigation of the precursors of antimicrobial compounds in kefir is currently necessary. *In vitro* antimicrobial activity against *Escherichia coli*, *Klebsiella pneumoniae*, *Pseudomonas aeruginosa*, *Enterococcus faecalis*, *Bacillus cereus*, *Bacillus subtilis*, and *Staphylococcus aureus* was attributed to bioactive peptides produced from *β*-casein, k-casein, *α*s1-casein, and *α*s2-casein present in the milk [64].

Kefiran has demonstrated an antimicrobial effect for *Streptococcus faecalis*, *Pseudomonas aeruginosa*, *Salmonella typhi*, *Bacillus subtilis*, *Bacillus cereus*, *Escherichia coli*, *Klebsiella pneumoniae*, *Staphylococcus aureus*, *Streptococcus faecalis*, and *Fusarium graminearum* [61, 69, 71]. In addition, kefiran also had action on fungi (*Aspergillus flavus* AH3) producing aflatoxins; a decrease from 100% to 33.3% of aflatoxin B1 production accompanied a decline in the mycelial dry weights [71]. Still, kefiran presented a more dramatic antimicrobial effect on *E. coli* than *S. aureus*, which can be attributed to the peptidoglycan in the cell wall of the latter. Peptidoglycan, in turn, hinders the diffusion of the antimicrobial through the cell. In addition, reports suggest that kefiran can reduce the concentration of antibiotics needed to obtain the antimicrobial effect, as illustrated by its synergism with ciprofloxacin [69]. It is interesting since extended administration of ciprofloxacin results in gastric and intestinal side effects [69].

Still, given the promising results of kefiran as a natural antimicrobial potential, its extraction methods from kefir have also been studied to maximize its antimicrobial effects. The ultrasound combined with hot water in the kefiran extraction process showed synergistic results about the antimicrobial activity compared to the kefiran extracted by the isolated methods [61]. *Lactobacillus kefiranofaciens* DN1 produced an EPS—composed of mannose, arabinose, glucose, galactose, and rhamnose—which at 0.3% demonstrated a bacteriostatic effect against *Listeria monocytogenes* and *Salmonella enteritidis*. In higher concentrations (1% and 2.5%), the bactericidal effect was obtained by completely inhibiting the growth of both microorganisms, being considered a new bioactive compound that can be used as a natural antimicrobial [65].

Organic acids are other antimicrobial compounds produced by kefir microorganisms. In this context, lactic acid was a bioactive compound associated with the antimicrobial effect of cow milk kefir and donkey milk kefir. Donkey milk kefir reduced *Klebsiella pneumoniae*, *Bacillus cereus*, and *Proteus mirabilis* by 8%, 37%, and 58% compared to the kanamycin antibiotic. In contrast, cow milk kefir decreased *B. cereus* by 12.9% while had the same impact on *Proteus mirabilis* compared to kanamycin, demonstrating the tremendous antimicrobial potential associated with lactic acid [60, 66]. However, there was no antimicrobial effect against *Pseudomonas aeruginosa* [17, 60]. Other organic acids present in kefir, such as acetic and pyruvic acid, also demonstrated an antimicrobial effect when administered together with lactic acid [14, 31, 70]. Kakisu et al. [31] identified that organic acids act against the germination of *B. cereus* spores in a dose-dependent manner. Using a higher concentration of kefir grains (5%) for the fermentation of milk reduced the pH value more significantly and more quickly, inhibiting the germination of the spores. This more significant lowering of the pH may be associated with more outstanding organic acid production in the medium and, consequently, a more significant antimicrobial effect. Moreover, kefir grains at 5% reduced in a range of 33.3% to 61.8% the output of NheA toxin by *B. cereus* [31].

Bacteriocin, a bioactive protein produced by microorganisms in kefir, demonstrated antimicrobial effects against several microorganisms. In this context, *Lactobacillus plantarum*, *Micrococcus luteus*, *Listeria monocytogenes*, *Salmonella enterica* serovar *Enteritidis*, *Staphylococcus aureus*, and *Bacillus cereus* were sensible when compared to the negative control (sterile deionized water). In contrast, *E. coli* proved resistant to the antimicrobial action of the bacteriocin. However, it is essential to note that *E. coli* was also resistant to the positive controls, nisin, and polylysine, which are natural antimicrobial peptides commonly employed as preservatives by the food industry. For *S. enterica*, antimicrobial effects similar to nisin were observed [40].

Some microorganisms in kefir produced fractions with antimicrobial activity against *Clostridium difficile*, *Pseudomonas putida*, *Pseudomonas aeruginosa*, and methicillin-resistant *Staphylococcus aureus* [59, 62, 63]. FK-1000 at 25 mg/mL inhibited the growth of *Pseudomonas aeruginosa* by 91%. However, even when in a lower concentration (0.25 mg/mL), FK-1000 presented a synergistic effect with streptomycin, potentiating five times the outcome of this antibiotic. It demonstrates the potential of FK-1000 for use in combination therapy. Another important aspect is that a 50 mg/mL concentration of FK-1000 was not toxic to human epithelial cells, increasing the relevance of this compound's use as a treatment [62].

In addition, thermolabile fraction greater than 10 kDa produced by *Lactococcus lactis* subsp. *lactis* CIDCA 8221 showed to inhibit the toxigenic effect of *C. difficile*; the fraction inhibited the interruption of the actin network and displacement of Vero cells caused by *C. difficile*. Also, a reduction in the formation of TcdA and TcdB toxins by *Clostridium* has been observed [59].

The proteolysis in the milk by kefir's microorganisms during fermentation leads to bioactive peptides with antimicrobial activity. F1 bioactive peptides reduced *E. coli* growth in a range of 33% to 57%. Consistently, a mixture of bioactive peptides from kefir had antimicrobial activity against several microorganisms such as *Klebsiella pneumoniae*, *Pseudomonas aeruginosa*, *Enterococcus faecalis*, *Bacillus cereus*, *Bacillus subtilis*, and *Staphylococcus aureus* [64, 68].

However, it is essential to note that the bioactive compounds from kefir appear to have different antimicrobial potencies. In general, when comparing interventions with similar target microorganisms (*E. coli*, *S. typhimurium*, and *S. aureus*), the concentration and time of intervention with organic acids were substantially higher (40 and 2 times, respectively) than for interventions with kefiran or bioactive peptides (Table 2). Therefore, kefiran and bioactive peptides may have more potent effects than organic acids, requiring lower concentrations for similar antimicrobial results. However, this premise needs further investigation.

*Lactobacillus plantarum*, a microorganism present in kefir grains, produces a biofilm that acts as an antimicrobial, inhibiting the growth of methicillin-resistant *Staphylococcus aureus* in the range of 1.4 to 30%. Medium's time, temperature, and aeration influenced the biofilm production by the kefir's microorganism, which may be related to cell maturation, enzymatic reactions, and activation expression of specific genes. Thus, external factors and factors related to the strain used can influence the antimicrobial activity associated with the production of biofilms by *Lactobacillus plantarum* [67].

Finally, the inhibitory effect of some microorganisms present in the kefir about pathogens may be associated with the presence of S-layer proteins on the outer surface of their cell membrane. Thus, the preincubation of *Lactobacillus kefir* with *S. enteritidis* allowed direct interaction between them through the S-layer proteins of *L. kefir*, reducing then the sites binding to enterocytes on the pathogen's cell membrane. In addition, S-layer proteins from *Lactobacillus kefir* strains also decreased the viability of *Salmonella enteritidis*. Interestingly, even S-layer proteins obtained from noncoaggregation strains, which do not interact with *Salmonella*, could interact with *S. enteritidis*. The conformation and the active groups present in the S-layer may differ between the protein isolated in the solution and the one present on the bacterium's surface (*Lactobacillus kefir*). Therefore, S-layer proteins have the potential to be used as a natural antimicrobial [72].

The meta-analysis for the antimicrobial category corroborates with the systematic review findings herein, as results show a statistically significant overall effect of kefir bioactive compounds against bacteria and fungi (Supplementary Figure S1). As the distinct outcomes' measurements differed between trials, we used the standardized mean differences (SMDs) to estimate the effects, whereas a negative SMD value indicates microbial reduction. Therefore, the standard mean differences (SMDs) were estimated for the overall antimicrobial effect (SMD (-1.35) (95% CI: -1.79–-0.91, *P* ≤ .001)) and separately for fungi (SMD (-7.18) (95% CI: -9.08–-5.28, *P* ≤ .001)), *Bacillus cereus* (SMD (-0.25) (95% CI: -1.06–0.56, *P* = 0.54)), *Escherichia coli* (SMD (-0.67) (95% CI: -2.26–0.92, *P* = 0.41)), *Klebsiella pneumoniae* (SMD (-1.79), (95% CI: -4.22–0.63, *P* = 0.15)), *Pseudomonas* spp. (SMD (-1.98), (95% CI: -3.48–-0.49, *P* = 0.009)), *Salmonella* spp. (SMD (-0.53), (95% CI: -1.12–0.06, *P* = 0.08)), and *Staphylococcus* spp. (SMD (-0.88), (95% CI: -1.81–0.06, *P* = 0.07)) as showed in the Supplementary Figure S1. In this context, kefir demonstrated a statistical significance concerning the overall antimicrobial effect, although the bioactive compounds from kefir only had significant effects against fungi and *Pseudomonas* spp. However, we can see a tendency to favor kefir treatments for all the studied microorganisms, although not statistically significant.

To assess the heterogeneity of the data, *I*^2^ tests for all nine analyses showed statistically significant considerable heterogeneity for fungi (*I*^2^ = 83%, *P* ≤ .001) and statistically significant moderate heterogeneity for overall antimicrobial effect (*I*^2^ = 72%, *P* ≤ .001), *Bacillus cereus* (*I*^2^ = 63%, *P* ≤ .001), *Escherichia coli* (*I*^2^ = 67%, *P* ≤ .001), *Klebsiella pneumoniae* (*I*^2^ = 64%, *P* = 0.004), *Pseudomonas* spp. (*I*^2^ = 61%, *P* = 0.0003), and *Salmonella* spp. (*I*^2^ = 68%, *P* ≤ .00001).

**Figure 2:**
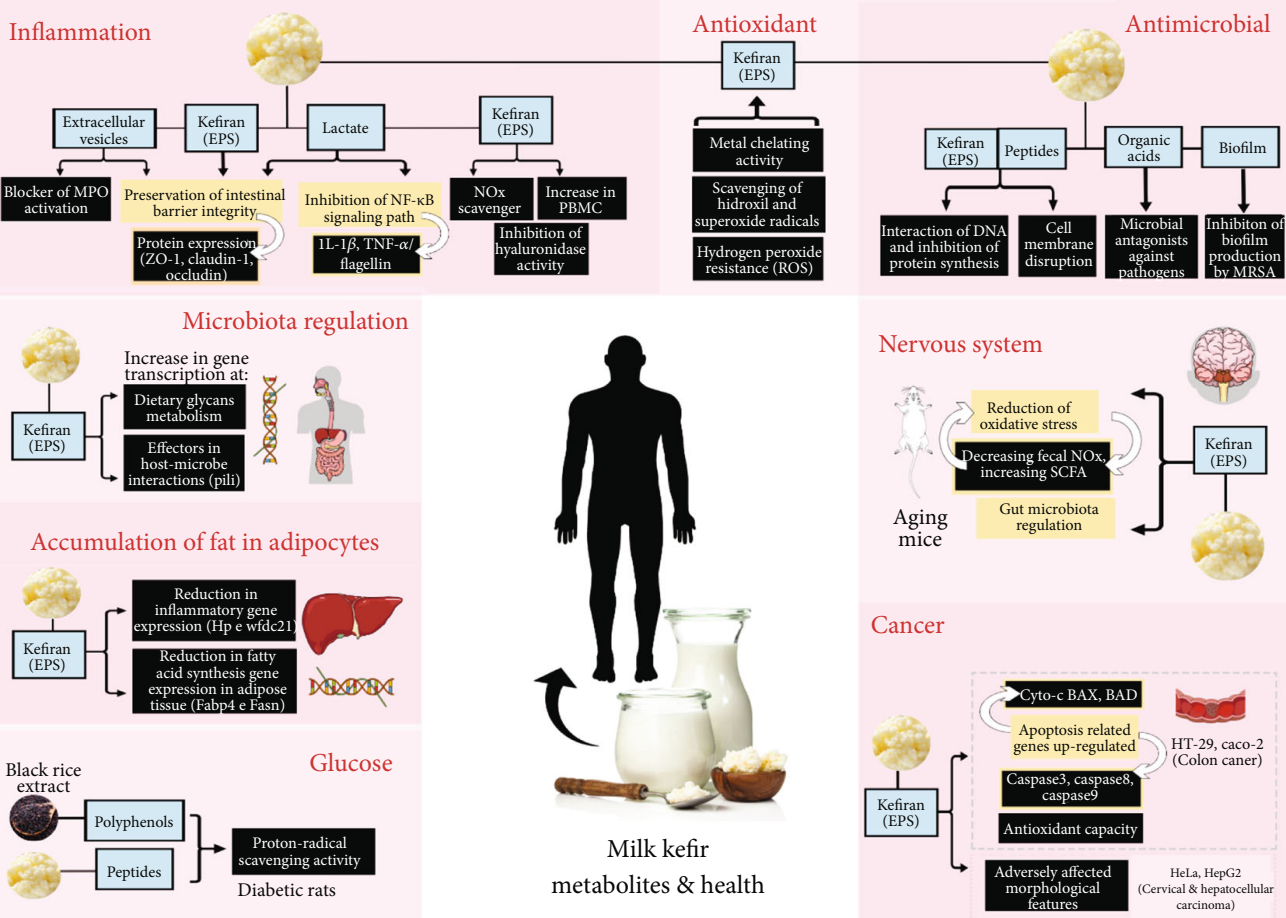
Mechanisms of action of bioactive compounds in milk kefir. EPS: exopolysaccharide; MPO: myeloperoxidase; ZO-1: zonula occludens-1; IL: interleukin; TNF*α*: tumor necrosis factor-*α*; NO: nitric oxide; PBMC: peripheral blood mononuclear cell; ROS: reactive oxygen species; MRSA: methicillin-resistant *Staphylococcus aureus*; Hp: haptoglobin-2; Wfdc21: Wfdc21 protein; Fabp4: fatty acid-binding protein 4; Fasn: fatty acid synthase; SCFA: short-chain fatty acid; Cyto-c: cytochrome c; BAX: BCL2-ssociated X protein; BAD: BCL2-associated agonist of cell death.

Despite the scarcity of studies investigating antimicrobials' mechanisms, these mechanisms appear diverse, presenting singularity for each type of bioactive compound. For bioactive peptides, the action occurs on the cell membrane and the pathogen's DNA, while organic acids reduce biological activity. Biofilms, in turn, inhibit the production of biofilm by the pathogenic microorganism (Figure 2). In this context, bioactive compounds form pores in the pathogen's membrane, which damages the integrity of the plasma membrane, increasing its permeability with consequent efflux of potassium ions, leakage of proteins, and nucleic acids. These compounds also were described bind to the genomic DNA of the pathogen [64, 68].

It is essential to highlight that although the *in vitro* and *in situ* studies imply an antimicrobial role of bioactive compounds from kefir, the absence of controlled *in vivo* studies is a gap in the literature that limits the assessment of the extent of this effect in physiological systems.

#### 3.4.2. Antioxidant Activity

The intervention with antioxidant molecules is crucial since they interact with free radicals, ending the chain chemical reaction and reducing the attack on proteins [28] and DNA [49] that would cause cell damage [84].

The main bioactive compounds identified with antioxidant activity from kefir were the EPS, including kefiran, although bioactive peptides and phenolic compounds were also described (Table 2). In addition, there have been few studies identifying the producing microorganisms of the compounds with antioxidant properties. However, the *Acetobacter*, *Leuconostoc*, *Bacillus*, and *Kazachstania* genera strains were reported (Table 2). Concerning the precursors in milk, there was an evident scarcity of their investigation; only one study identified the precursors of bioactive peptides as being *β*-casein, k-casein, *α*s1-casein, and *α*s2-casein [64].

The antioxidant activity of kefiran *in vitro* was 8.47 *μ*g/mL and 4.44 *μ*g/mL, for 1% and 0.5% concentrations, respectively, as measured by reducing power activity in ascorbic acid equivalent capacity (AAEC). Interestingly, the hyaluronic acid used in gold standard viscosupplementation treatment did not demonstrate reducing power at similar concentrations to those employed for kefiran. It shows the antioxidant potential of the compound isolated from kefir [54]. The scavenging of free radicals and ferric ion reduction represented 25% to 85% and 37% to 84%, respectively, of vitamin C activity, which is considered a positive control for these activities. The relatively low concentrations (0.005% to 0.08%) tested of kefiran can justify its inferior effect compared to vitamin C [61].

The antitoxic activity of the EPS produced by microorganisms in the kefir promoted the protection of biological molecules from oxidation. Inline, low concentrations of EPS (0.05% to 0.25%) could protect BSA protein from oxidation induced by APPH (2,20-azobis(2-methypropionamidine) dihydrochloride). The protein oxidation-decrease ranged from 31% to 96%. Interestingly, this protection was higher than observed in the negative control (protein without induced oxidation) [28]. In addition, the antioxidant capacity *in situ* related to EPS was higher 8.43% after 24 hours of fermentation [74]. The ability to eliminate the DPPH (1,1-diphenyl-2-picrilhidrazil) free radical, turn in, increased from 10% to 20%. This fact can be attributed to the metabolic activity of kefir microorganisms during fermentation, leading to the production and accumulation of EPS. Thus, studies have found that the EPS present in kefir demonstrates potential as an antioxidant agent [73]. The EPS also confers resistance to hydrogen peroxide, completely reversing the detrimental effect of this compound on the cell growth of microorganisms present in the kefir. Consistent, cells without EPS significantly lose resistance to hydrogen peroxide [73].

Smaller fractions present in kefir, such as bioactive peptides, also demonstrated an antioxidant effect. An enhancement in scavenging ability of oxidized 2,2′-zino-bis (3-ethylbenzothiazoline-6-sulfonic acid) (ABTS) ranged from 2% to 25.6% for sheep's milk kefir and 35.8% for cow's milk kefir both compared to control [14, 64]. A significant improvement (111.6%) in oxygen radical absorbance capacity (ORAC) for cow's milk kefir confirmed the antioxidant activity of bioactive peptides [14]. We believe that the observed difference between the antioxidant activity of sheep's milk kefir and cow's milk kefir may be associated with the difference in composition between milk, especially about the protein content and its design which are precursors of bioactive peptides. This finding highlights the relevance of identifying the precursors of bioactive compounds in milk to increase its functional potential.

The fermentative process of milk by kefir grains showed to elevate its antioxidant activity by synthesizing phenolic compounds. In artisanal kefir, the antioxidant capacity was increased up to 120% when measured by ABTS and up to 40% by ORAC [75]. In contrast, for commercial kefir, the antioxidant capacity by ABTS reduced, while that by FRAP—ferric antioxidant power—increased [85]. Thus, phenolic compounds produced by starter culture appear to exert antioxidant action through their reducing power but are ineffective as to the scavenging ability of oxidized compounds. Still, the DPPH scavenging varied according to the type of kefir, reducing in ewe kefir and increasing in cow kefir compared to control [85]. Therefore, according to the matrix, the profile of phenolic compounds can influence the *in situ* radical scavenging ability. Indeed, Satir and Guzel-Seydim [75] observed increased antioxidant activity in the presence of gallic acid, catechin, epicatechin, caffeic acid, p-coumaric acid, chlorogenic acid, ferulic acid, and photocatechuic acid. Phenolic compounds resulting from the metabolic activity of kefir microorganisms can improve up to 120% of the *in situ* antioxidant capacity during fermentation [85].

The intervention times were more remarkable for bioactive peptides than EPS and phenolic compounds for similar methodologies (Table 2). This finding suggests that the peptides need a longer time to exert significant antioxidant activity. On the other hand, phenolic compounds required concentrations up to 3 times higher than other bioactive compounds (Table 2).

Consistent with the described findings, the meta-analysis showed an antioxidant tendency of the kefir bioactive compounds. However, it was not considered statistically significant compared to control treatments, even though we observed a narrow confidence interval. It resulted in a standard mean difference (SMD) of (SMD (-0.83) (95% CI: -1.65–0.00, *P* = 0.05)) (Supplementary Figure S2). Besides, the outcomes of the studies showed a significant substantial heterogeneity (*I*^2^ = 84%, *P* ≤ .001), which may have contributed to the lack of significance.

Although there is a scarcity of description of the mechanisms of action in the literature, EPS seems to exercise effect through metal chelating activity and sequestering activity of hydroxyl and superoxide radicals, with consequent resistance to the hydrogen peroxide. In contrast, the antioxidant activity of bioactive peptides cannot be attributed to the ion chelating ability [14].

As reported previously for antimicrobial effect, the absence of controlled *in vivo* experiments evaluating the antioxidant potential of bioactive compounds from kefir is a gap in the literature that limits assessing the extent of this effect in physiological systems.

#### 3.4.3. Modulation of the Intestinal Microbiota

Maintaining the intestinal microbiota in symbiosis with the host is essential for human health since it favors the integrity of the intestinal barrier, the balance of the immune system, and controlling inflammatory processes. Therefore, the search for probiotics or bioactive compounds which favor the modulation of the intestinal microbiota has been extensively studied in the recent literature.

Exopolysaccharides (EPSs), including kefiran, were the predominant described bioactive compounds capable of modulating the intestinal microbiota *in vitro* and *in vivo* models (Table 2). However, there is a lack of studies identifying the precursors of these bioactive compounds. In addition, the scarcity of studies determining the microorganisms that produce them in milk highlights a gap in the literature. Interestingly, the concentration and intervention time using bioactive peptides was considerably higher than those with EPS for *in vivo* animal models; the concentration employed of peptides was up to 2.5-fold greater. The intervention time was up to 2.6-fold longer compared to EPS use (Table 2). It suggests that EPS can be a bioactive compound more potent for gut modulation. However, this premise needs further investigation.

EPS produced by *L. paracasei* CIDCA 8339 and CIDCA 83124 in kefir demonstrated modifying the microbiota present in infant fecal samples and, consequently, changing the short-chain fatty acid profile (SCFA). The butyric and propionic acids produced are compounds with biological activity associated with health benefits. Among these benefits stand out are as follows: strengthening the intestinal epithelial barrier and inhibiting the cholesterol synthesis at the liver. Still, the expression of leptin, YY polypeptide (PYY), and glucagon-like peptide 1 (GLP-1) promoted by them regulate the lipogenesis in adipose tissue and the appetite [79].

Considering the phylum level, the use of EPS and bioactive peptides commonly improved *Firmicutes* to the detriment of *Bacteroidetes*, as demonstrated in Table 2. Regarding the genus level, a reduction in microorganisms associated with pathogenicities, such as *Klebsiella* and *Escherichia,* has been reported, demonstrating the potential of EPS in contributing to a healthy intestinal microbiota [79]. The concomitant decrease in *Rikenellaceae* is also a promoter of the health of colon epithelial tissue [81]. In contrast, EPS favored *Victivallis*, *Acidaminococcus*, *Comamonas*, and the *Ruminococcaceae* family [79, 81]. Selecting certain species of the *Acidaminococcus* genus may be responsible for increasing organic acids such as propionate and butyrate, which are beneficial in intestinal levels.

On the other hand, the modulation of other genera also considered beneficial to health, such as *Lactobacillus* and *Bifidobacterium*, depended on the bioactive compound used during the intervention (Table 2). Thus, *Lactobacillus* and *Bifidobacterium* were not favored by EPSs 8339 and 83124 in Bengoa et al. [79]. In contrast, in the study of Xing et al. [81], using adult male mice, EPS produced by *Lactobacillus kefiranofaciens* XL10 provided growth of 14.59% for *Lactobacillaceae* and up to 0.59% for *Bifidobacteriaceae*. Growth of up to 17% in *Bifidobacterium* in adult female mice was also observed when using kefiran, although no change was observed in the *Lactobacillus* population [82]. *In vitro*, 0.3% kefiran elevated a *Bifidobacterium bifidum* PRL2010 population up to 5.8 × 10^8^ CFU/mL. In contrast, there was not *Bifidobacteria* population growth in the control (medium without carbon source). This fact indicates that the target microorganisms should be considered in deciding the most appropriate bioactive compound for intestinal modulation in each intervention.

Interestingly, EPS was more potent in promoting intestinal microbiota diversity than inulin, a prebiotic commonly used by the food industry. Still, EPS led to a different short-chain fatty acid profile, increasing the butyrate content and benefiting the gut microbial population more than inulin [79]. Kefiran also proved to be a better source of carbon than glucose for the growth of *Bifidobacterium bifidum* PRL2010; kefiran increased the development of the strain by 20 to 700% compared to the use of glucose [41].

Bioactive peptides, other bioactive compounds present in kefir, were also related to changes in the intestinal microbiota. The oral administration of peptides to female mice partially reversed the detrimental effect on the intestinal microbiota caused by oophorectomy; the peptides from kefir reduced 46% of *Parasutterella* and 39% of *Streptococcus*. Bacteria potentially pathogenic belonging to genera *Klebsiella* and *Escherichia* were also decreased. *Romboutsia*, together with *Streptococcus*, has been linked to obesity and presented an 86% reduction (Table 2). Bioactive peptides from kefir elevated the *Alloprevotella* population by more than 30% by reducing oophorectomy-induced renal fat accumulation. *Ruminococcus*-1, SCFA producer bacteria, increased in ovariectomized mice, subsequently decreasing with bioactive peptides' administration. It is essential to highlight that the growth of butyrate-producing bacteria after estrogen deficiency in ovariectomized mice can negatively lead to a detrimental accumulation of SCFAs in the intestine. In contrast, bioactive peptides have not been able to restore the reduction in the *Deferribacteres* phylum caused by oophorectomy; the decrease of *Deferribacteres* is related to the detriment of vitamins and amino acid metabolism [80].

Thus, bioactive peptides can enrich beneficial bacteria and decreasing potentially harmful pathogens in the gut of ovariectomized females [80]. Still, these findings reveal that further studies, analyzing the relationship between intestinal microbiota and estrogen deficiency and the role of the bioactive peptides from kefir in this relationship, are necessary for a better understanding.

Concerning the richness and diversity of microbiota, interestingly, it is not affected by bioactive peptides in ovariectomized mice. However, peptides significantly improve intestinal microbiota diversity compared to control without ovariectomized procedure [80].

Regarding meta-analysis, treatments with bioactive compounds did not present statistically significant effects on gut microbiota modulation compared to control treatments (SMD (-0.39) (95% CI: -1.32–0.54, *P* = 0.41)) (Supplementary Figure S3). However, we can notice a tendency that favors bioactive efficacy, consistent with our findings. This analysis presented a statistically significant moderate heterogeneity (*I*^2^ = 58%, *P* < .001), which may have contributed at least partially to the lack of significance.

Finally, most studies failed to evaluate the mechanisms of action of the bioactive compounds on gut microbiota modulation, representing a gap in the literature. However, enhanced transcription of genes involved in the microbe-host interaction was proposed for the kefiran action.

#### 3.4.4. Immune Response Modulation

Bioactive compounds from kefir exerted an anti- or proinflammatory impact depending on the model's presence or absence of inflammatory insult. Thus, they exerted an inhibitory effect in inflammatory diseases' models, while they had an immunostimulatory effect for models without inflammatory insult (Table 2).

The predominantly studied inflammatory disease model was that of acute colitis, both *in vitro* and *in vivo*. For colitis, mainly EPS and extracellular vesicles, but also lactate, they have been described to have an anti-inflammatory role against a variety of acute inflammatory insults: DSS (dextran sulfate sodium), TNF*α*, FliC (flagellin), IL-1*β*, and TNBS (2,4,6-trinitrobenzene sulfonic acid). For chronic colitis, extracellular vesicles presented an anti-inflammatory effect against piroxicam. Kefiran, in turn, had an inhibitory effect on cotton-induced granuloma in rats (Table 2). The *L. kefirgranum*, *L. kefir*, *L. kefiranofaciens*, and *L. paracasei* species were responsible for producing these bioactive compounds. Therefore, the *Lactobacillus* genus seems relevant for making anti-inflammatory compounds in the kefir [13, 50, 53]. Galactose and glucose, and to a lesser extent, mannose, arabinose, and rhamnose, were the significant precursors of the polysaccharide component of the bioactive compounds [55].

Extracellular vesicles at 100 *μ*g/mL from *L. kefirgranum* reduced the gene expression of IL-2, IL-8, and TNF*α* proinflammatory cytokines by 58, 64, and 67%, respectively, in Caco-2 cells for DSS-induced acute colitis model [50]. Extracellular vesicles (1 × 10^9^ particles/mL) also inhibited TNF*α*-induced colitis. They reduced the expression and secretion of IL-8 by 65 and 96%, respectively, in the Caco-2 cell line. Interestingly, the extracellular vesicles were just as effective as budesonide [53], a glucocorticoid steroid commonly used to treat Crohn's disease (inflammatory bowel disease) [86]. Still, treatment of Caco-2 cells with extracellular vesicles showed a longer intervention time than treatments with EPS or lactate. In addition, the effect observed with preincubation of cells with EPS or lactate (Table 2) indicates the potential of these compounds as preventive agents of intestinal inflammation. However, further studies exploring pre- and postincubation concerning the inflammatory insult must be conducted to elucidate these bioactive agents' preventive and therapeutic potentials.

In mice, oral administration of extracellular vesicles could mitigate acute and chronic colitis, corroborating the previous findings *in vitro*. For DSS-induced acute colitis, both high and low dosages of vesicles prevented weight loss in mice by up to 16% and reduced damage to colon tissue by up to 63%. However, only the highest dosage (3 mg/kg bw) reduced colon atrophy by 29.6%. Similarly, only the highest dosage mitigated colon atrophy by 14.3% for chronic colitis aggravated by piroxicam. Nevertheless, both dosages reduced colon histological damage by up to 85%.

In contrast to acute colitis, ingestion of extracellular vesicles was ineffective in preventing weight loss in chronic colitis [50]. Therefore, due to the broader effects obtained for 3 mg/kg bw, the high dosage seemed more effective for chronic and acute colitis treatment. Vesicles' administration against TNBS-induced acute colitis also effectively prevented the mouse weight loss by up to 12.5% at 3 × 10^8^ and 3 × 10^10^ vesicles/head. Moreover, the administration reduced rectal bleeding and diarrheal condition severity by 75 and 91%, respectively. Damage to colon tissue, in turn, was decreased by up to 85% [53]. Therapy with vesicles from *Lactobacillus* of kefir was more effective than the prednisolone drug (2 mg/kg) in preventing weight loss, the severity of rectal bleeding, and diarrheal conditions well as in mitigating colon histological damage [53]. Prednisolone is an anti-inflammatory steroid used to treat inflammation in colitis and Crohn's disease; however, it does not prevent recurrence of the disease, in addition to having several side effects [87, 88]. Therefore, treatment with bioactive compounds from kefir would be promising both for effectiveness and reduced side effects.

Suspensions of EPS-producer *Lactobacillus paracasei* (OD_590_ = 0.25) inhibited by up to 55% the induction of the CCL20 proinflammatory promoter in Caco-2 cells for flagellin-induced acute colitis model. *Lactobacillus paracasei* CIDCA 8339 strain showed more dramatic anti-inflammatory potential than the other tested strains [13], which indicates that the functional potential of the EPS is strain-dependent. Similarly, lactate at 100 mM inhibited by 78, 80, and 42% the CCL20 promoter induction by flagellin, IL-1*β*, and TNF*α*, respectively, in Caco-2 cells. Inline, human intestinal epithelial cells express the lactate receptor. Still, lactate solution and supernatant from kefir with corresponding lactate concentration showed similar inhibitory effects on Caco-2 cells [56], indicating that the kefir matrix does not reduce the impact of this bioactive compound. *In vivo*, oral administration of kefiran-rich kefir supernatant (1 mL/day) was responsible for reducing the weight of cotton-induced abdominal granulomas by 44% in rats. Kefir was as effective as dexamethasone (0.2 mg/kg) in reducing these granulomas [16]. Dexamethasone is a corticosteroid medication used as a primary option in the treatment of granulomas [89]. This evidence reinforces the anti-inflammatory potential of bioactive compounds from kefir.

Therefore, in general, bioactive agents inhibited the expression of proinflammatory cytokines and the activation of the CCL20 promoter for *in vitro* inflammatory models with Caco-2 cells. In *in vivo* colitis models, bioactive compounds reduced weight loss, atrophy, and colon histological damage.

The compounds displayed anti-inflammatory action mechanisms by inhibiting the NF-*κ*B pathway in the Caco-2 cells and the colon mucosa due to the expression of the I*κ*B*α* inhibitor [50, 53, 56]. In addition, bioactive agents promoted the integrity of the intestinal barrier, increasing the expression of occludin, ZO-1, and claudin-1 occlusion proteins [50]. Additional anti-inflammatory mechanisms proposed for EPS were nitric oxide radical scavenging ability [54] and inhibition of hyaluronidase activity in cell-free *in vitro* systems [55]. Hydrolysis of the extracellular matrix by hyaluronidase releases compounds, like hyaluronan, throughout inflammatory pathologies [90]. For extracellular vesicles, blocking myeloperoxidase activation in the mouse plasma has also been reported [53]. Oxidative stress in inflammatory bowel disease activates inflammatory cells, such as neutrophils, whose myeloperoxidase catalyzes the production of reactive oxygen species [91]. In this scenario, extracellular vesicles were as effective as the prednisolone drug in inhibiting myeloperoxidase [53]. Thus, the evidence suggests that bioactive compounds from kefir may play a decisive anti-inflammatory role.

However, EPS, including kefiran, can also have the opposite effect, acting as immunostimulants, in cases where there is no inflammatory insult (Table 2); this role was also corroborated in an *in vivo* model [58]. Their precursors in milk were glucose and galactose [51, 52]. In a minority way, bioactive peptides have also been reported as immunostimulants [57]. The *Lactobacillus* genus was relevant to produce these immunostimulants, especially the *L. helveticus*, *L. pentosus*, and *L. kefiranofaciens* species.

For EPS, although the intervention time has been similar for both a pro- and anti-inflammatory effect assay, the EPS concentration employed was dramatically higher; *in vitro*, the concentration for immunostimulating varied from 50 to 5000 *μ*g/mL [4, 46, 51], while for inhibition, it reached the maximum of 100 *μ*g/mL [50]. *In vivo*, 100 mg/kg bw was administered orally for immunostimulating [58], while to inhibit the immune response, the concentration ranged from 0.03 to 3 mg/kg bw [50]. This fact suggests that the concentration of EPS is a significant factor in determining the role that it will play on the immune system. In line, EPS has been reported to stimulate or inhibit the secretion of TNF*α*, IL-10, and IL-6 by *in vitro* murine macrophages, depending on the concentration tested [51, 52]. Thus, it appears that EPS can act by different cell signaling pathways, according to its concentration. However, the immunostimulatory mechanisms of action still need to be studied.

*In vitro*, the immunostimulatory role of EPS and bioactive peptides has been demonstrated in the macrophage's cell line and primary culture, in addition to human peripheral blood mononuclear cells (PBMCs) (Table 2). R-5-EPS and R-17-EPS at 50-400 *μ*g/mL stimulated proliferation, phagocytosis, phosphatase activity, IL-6 secretion, and NO production by RAW264.7 murine macrophage cells. Still, in the concentration range of 100 to 200 *μ*g/mL, they stimulated the secretion of TNF*α*, IL-1*β*, and IL-10. EPSs were as efficient as lipopolysaccharides in promoting cell proliferation, phagocytosis, and cytokine secretion by macrophages [51, 52]. Kefiran at 1000 to 5000 *μ*g/mL increased IL-6 secretion, and concentrations from 2000 to 4000 *μ*g/mL stimulated cell proliferation of human PBMC culture by up to 200% after 24 h [46]. Bioactive peptide from *L. kefiranofaciens*, turn on, improved secretion of TNF*α*, IL-1*β*, IL-6, and IL-12 by 1000, 700, 1300, and 3000% by murine peritoneal macrophage culture. However, peptides from different microbial strains showed differences in immunostimulatory capacity [57], suggesting that the functional potential of the peptide is strain-dependent. In addition, bioactive peptide acted via the TLR2 receptor [57]; Toll-like receptor 2 enables macrophages to recognize microbial ligands, thereby promoting inflammation [92].

Consistently, oral administration of kefiran (100 mg/kg bw) to healthy mice for up to 7 days enhanced IgA, IL-10, IL-6, and IL-12 in the mucosa of the small intestine, as well as IL-4 and IL-12 in the fluid of the small intestine. In serum, kefiran increased IL-4, IL-6, IL-10, and IFN. However, broader immunostimulation occurred in the large intestine, increasing IgA, IgG, IL-4, IL-10, IL-6, INF, and TNF content. The most evident stimulatory activity in the large intestine has been attributed to the kefiran fermentation by intestinal microbiota [58]. Therefore, it appears that bioactivity may vary according to the biochemical transformations that these molecules undergo throughout the digestive process.

Finally, it is essential to highlight that immune stimulation can be interesting for a better prognosis of infectious conditions [93] and stimulating immunoglobulin production after vaccination [94]. Thus, the concentration and environmental context (presence or absence of inflammatory insult) are relevant factors to be considered according to the purpose of administering the bioactive compound.

Meta-analysis results corroborated the benefits of kefir bioactive compounds on immune modulation since the findings indicated that treatments had significant immune-modulatory activity compared to control (Supplementary Figure S4). The estimated (SMD) was (SMD (-1.17) (95% CI: -1.47–-0.87, and *P* ≤ .001)). Heterogeneity was statistically significant moderate for this analysis (*I*^2^ = 67%, *P* ≤ .001).

#### 3.4.5. Anticancer Effect

Cancer is a term that encompasses more than 100 different types of malignancies that have in typical disordered cell growth, which can metastasize [95]. However, although human *in vitro* models have reported anticancer effects of bioactive compounds from kefir on breast, colon, cervical and hepatocellular cancers, the lack of *in vivo* studies limits the understanding of the extent of their anticancer effect in organisms. Among the bioactive ones, EPS, including kefiran, had a broader impact, covering different types of cancer: breast, colon, cervical, and hepatocellular cancers (Table 2). The main precursors of EPS in the milk were glucose and galactose [47]. The *Lactobacillus* genus seemed relevant for EPS production with an anticancer effect on kefir (Table 2).

Kefiran reduced up to 45% of MCF7 breast cancer cell viability after 48 h of intervention, losing effect under the highest tested concentration of 4 mg/mL. However, efficiency decreased to 15.6% after 72 h, without effect at the lowest tested concentration of 0.5 mg/mL [46]. For HepG2 hepatocellular carcinoma cells, kefiran reduced their viability by up to 82% from the minimum concentration of 250 *μ*g/mL. In contrast, for HeLa cervical carcinoma cells, kefiran reduced the viability by up to 72% in a dose-dependent manner [48]. Similarly, EPS MRS101 presented a dose-dependent effect on the viability of HT-29 colon cancer cells, reducing the number of viable cells by up to 55.9% after 24 h [47]. Therefore, the EPS concentration and the intervention time seem to be decisive for obtaining or not the anticarcinogenic effect. However, it is essential to note that developmental toxicity was observed in zebrafish embryos for kefiran concentrations above 100 *μ*g/mL so that kefiran at 1000 *μ*g/mL reaches 80% mortality [48]. Inline, 10^−3^ to 3 *μ*g/mL of EPS showed no toxicity on typical Vero cell culture [55]. Therefore, EPS can affect the growth of normal tissues depending on the concentration used, so this factor should be considered in future clinical applications.

The proposed mechanism of action for EPS was the upregulation of the apoptotic genes Cyto-c (cytochrome c), BAD (BCL2-associated agonist of cell death), BAX (BCL2-associated X protein), and caspases 3, 8, and 9; the increase in the expression of these genes ranged from 15 to 120%. In contrast, EPS downregulated by 70% the BCL2 gene, which is involved with the oxidative stability of the mitochondria [47]. Such changes in gene expression may be related to the adverse effects on the morphology of cancer cells. Loss of adherence capacity and formation of intracellular vacuoles were the more common changes resulting in cell death [48].

On the other hand, bioactive peptides have been reported only in an estrogen-sensitive breast cancer model. In addition, they showed a longer intervention time than other bioactive compounds described (Table 2). However, peptides appear to have lower toxicity as an advantage to EPS since peptides from kefir did not exert an antiproliferative effect on normal human mammary epithelial cells at doses of 0.31 to 10% (v/v). On the other hand, on those exact dosages, peptides reduced dose-dependent manner by up to 88% the MCF7-E3 human breast cancer estrogen-sensitive cell number [15], which indicates the specificity of this bioactive compound. Still, yogurt extracts showed dramatically lower efficiency than kefir extracts, in addition to presenting toxicity on normal human epithelial cells from a 5% (v/v) concentration. Nonfermented milk, unlike, had a negative effect, stimulating the proliferation of MCF7-E3 cancer cells [15]. These findings corroborate the anticarcinogenic specificity of peptides from kefir.

Although the anticancer effect of organic acids has not been directly tested, lactic and acetic acids present in kefir reduced fecal water-induced-DNA damage in HT-29 cells. Cultivation with fecal water for 30 min increased by 36% the DNA damage in HT-29 cells, being this effect more toxic than that caused by hydrogen peroxide [49]. However, fecal water-induced-DNA damage was inhibited with kefir supernatant by 20%. Thus, organic acids can have a preventive effect against colon cancer, attributed to their antioxidant activity. Inline, kefir presented antioxidant capacity (Trolox equivalent) 78.6% greater than unfermented milk [49], corroborating the role of organic acids produced during fermentation in protecting DNA.

Finally, the HT-29 colon cancer cell line [47, 49] appeared to be more sensitive to EPS than the MCF7 breast cancer cell line [46]. For the latter, the concentration reported for the assay was ten times greater (Table 2). Thus, sensitivity to the bioactive compounds from kefir appears to be dependent on cell type.

Validating our findings, treatments with bioactive compounds presented a significant anticarcinogenic effect, as the estimated (SMD) for overall anticancer effect was (SMD (-2.44) (95% CI: -3.41–-1.47, *P* ≤ .001)) (Supplementary Figure S5). The data were homogeneous (*I*^2^ = 46%, *P* ≤ 0.0006), which means good consistency among studies.

#### 3.4.6. Plasma Glucose

Diabetes mellitus is a chronic glucose metabolism disorder with severe clinical consequences, such as retinopathy, nephropathy, and stroke [96]. Type 2 diabetes mellitus is a metabolic disorder marked by the rise in blood glucose due to a decrease in insulin secretion by pancreatic *β*-cells and insulin resistance. Additionally, the increase in the prevalence of diabetes mellitus worldwide in recent decades is associated with the rise in the prevalence of obesity in the population [96].

In this scenario, bioactive compounds in kefir were tested on glucose metabolism in obesity and diabetes murine models *in vivo* (Table 2). Oral administration (10 mL/kg bw) of a mixture of EPS-producer *Leuconostoc mesenteroides* LMDH4 and *Lactobacillus kefiri* LKDH5 during eight weeks did not affect the plasma glucose level in diet-induced obese mice. However, this treatment effectively reduced adipocyte tissue weight by 36%, in addition to downregulating the proinflammatory and fatty acid synthesis gene expression in the adipocytes [76]. This fact indicates that the regulation of fatty acid metabolism in adipose tissue is not a determining factor in promoting a significant change in plasma glucose.

In contrast, administration of a mixture of 5 to 20 mL of kefir with black rice extract—1 : 1/kg bw—during four weeks enhanced by up to 199% and 2330% of the Langerhans islet in the pancreas and insulin-positive *β*-cells, respectively, in diabetic rats. The concentration from 10 mL/kg bw completely reversed the pancreatic damage induced by STZ-NA (Streptozotocin-nicotinamide), achieving a similar effect to the glibenclamide [78]. It is an antidiabetic drug of the second-generation sulfonylureas class that reduces blood glucose by increasing insulin secretion from pancreatic *β*-cells [97]. The effect was attributed to the proton-radical scavenging activity from alcohol and phenolic compounds present in the beverage [78]. This finding demonstrates that the antioxidative capacity must be a significant factor in promoting the homeostasis of insulin production by the pancreas. However, as the study did not test a formulation without adding black rice extract, it is impossible to determine how much of this positive effect on *β*-cells can be attributed to the kefir alone. Indeed, the addition of black rice extract increased by 56.8% the antioxidant capacity of the beverage compared to the kefir alone, as measured by DPPH [78].

Therefore, the current findings in the literature on the effect of bioactive compounds of kefir on glucose metabolism are inconclusive, so further preclinical studies with pure kefir drink or with bioactive compounds isolated from it are urgently needed.

#### 3.4.7. Effect on Serum Cholesterol and Accumulation of Fat in the Adipose Tissue

Cardiovascular disease (CVD) is one of the major leading causes of morbidity and mortality worldwide. However, deaths by CVD are attributable to manageable risk factors, the main ones being high total serum cholesterol, high blood pressure, and smoking [98]. In addition, hypercholesterolemia is one of the risk conditions related to obesity [99]. In this scenario, reducing total serum cholesterol and fat accumulation in adipocytes is one of the strategies used to prevent CVD. However, pharmacological drugs to treat obesity reportedly cause several side effects related to blood pressure, hepatic failure, pancreatitis, and headaches [100]. Therefore, research on natural compounds for the treatment of obesity has been encouraged.

Among the bioactive compounds from kefir, only EPS has been associated with lowering adipocyte fat and reducing *in vitro* cholesterol. *Lactococcus lactis* WH-C1, *Lactobacillus kefiri*, and *Leuconostoc mesenteroides* were the microorganisms identified as producers of these EPSs in the kefir [77]. However, the lack of investigation of their precursors in the milk shows a gap in the literature (Table 2).

EPS-producer *Lactococcus lactis* WH-C1 (4%, v/v) isolated from Tiber kefir grain could remove up to 31.23% of cholesterol from culture medium, demonstrating a potential hypocholesterolemic property *in vitro* [77]. In contrast, EPS was ineffective on an *in vivo* experimental model; supplementation for eight weeks with a mixture of EPS-producer heat-killed lactic acid bacteria (HLAB) isolated from kefir did not change the lipid profile in obese mice. Thus, serum HDL, LDL, total cholesterol, and triglycerides were not affected in diet-induced obese mice [76]. The lack of effect on serum cholesterol indicates that the concentration of EPS-producing microorganisms employed (1.1 × 10^10^ CFU/mL) may have been insufficient to achieve significant changes in the cholesterol level *in vivo*. It must be taken into account that microorganisms became unable to continue the EPS production when they died from heating. Indeed, the greater the growth of the EPS-producing microorganism, the greater the cholesterol removal rate from the medium was demonstrated *in vitro* [77].

Similarly, kefir supplementation, which did not lead to a modulation of gut microbiota, is associated with low propionic acid production in the colon, which is insufficient to promote changes in the serum cholesterol level [101]. Also, the diet's inclusion of extract rich in polyphenol (wine grape seed flour -2.5%, v/v) did not alter the serum cholesterol levels [76]. Thus, antioxidative capacity may not be a significant factor in reducing blood cholesterol levels.

On the other hand, the EPS ability to reduce the fat accumulation in the adipocytes was confirmed *in vitro* and *in vivo* animal models (Table 2). EPS isolated from strains of *Lactobacillus kefiri* and *Leuconostoc mesenteroides* showed inhibition (up to 28%) of lipid accumulation in 3T3-L1 adipocytes *in vitro*; this inhibition, in general, occurred in a dose-dependent manner [76]. In addition, the minimum concentration of EPS to obtain the effect appeared to be strain-dependent since only EPS from *L. mesenteroides* LMDH4 presented an effect at 0.01 mg/mL. At the same time, those from *L. kefiri* LKDH3 and *L. mesenteroides* LMDH6 affected the fat accumulation only at the highest concentration tested (0.2 mg/mL). In contrast, EPS from *L. mesenteroides* LMDH8 and LMDH9 did not act even at the highest concentration tried (Table 2). This finding implies that the functional potential of EPS on fat accumulation varies according to its microbial origin.

About *in vivo* animal experiment, a diet supplemented with EPS-producer HLAB during eight weeks reduced by 36% the adipose tissue weight in C57BL/6J mice fed a high-fat and high-fructose diet [76]. This way, it suggests that EPS from kefir's microorganisms can be a functional ingredient capable of being used in obesity cases. EPS performance occurred through action on adipose tissue; expression of *Wdfc21* and *Hp* proinflammatory genes was lowered by 56% and 57%, respectively, in the adipose tissue compared to the control group. Also, the expression of genes related to the synthesis of fatty acids (*Fabp4* and *Fsan*) was downregulated by 55% and 43%, respectively, in the adipose tissue of the HLAB group. *Fabp4* encodes fatty acid-binding protein 4 (Fabp4), while *Fsan* encodes fatty acid synthase. It demonstrates that EPS has roles in anti-inflammation and inhibiting the fatty acid synthesis in adipocytes. However, these effects on the *Wdfc21*, *Hp*, *Fabp4*, and *Fsan* genes did not result in serum cholesterol changes, as its level has not been altered by treatment with HLAB.

Interestingly, supplementation of the diet with HLAB in conjunction with wine grape seed flour (2.5%), which is rich in polyphenol, potentiated the effect of reducing fat accumulation by 25% [76]. Therefore, antioxidant activity may be a relevant factor for reducing fat accumulation and can enhance the impact of EPS on adipocytes. Consequently, the synergistic treatment was the only one capable of significantly reducing the serum triglyceride.

#### 3.4.8. Antihypertensive Effect

Hypertension is a chronic disease that leads to an increase in blood pressure [102]. Angiotensin-converting enzyme (ACE) is a key component of the renin-angiotensin system as part of the homeostatic mechanism to maintain adequate blood pressure levels in mammals. Conversion of ACE 1 to ACE 2 (an enzyme with a vasoconstrictor effect) is a common mechanism to regulate blood pressure [103]. But in abnormal conditions, this constriction can cause high blood pressure and increase the work required for the heart to pump blood into the body's main arteries [104]. In this way, inhibition in the conversion of ACE 1 to ACE 2 plays a role in managing hypertension.

As the systematic review selected only one article, it was the major limitation to a consistent understanding of the effect of bioactive compounds from kefir on blood pressure. Still, this study did not investigate the microorganism that produces the bioactive compound, its mechanism of action, nor the precursor of this bioactive compound in milk. In addition, the model studied was *in situ*. The absence of an investigation *in vivo* limits the understanding of the functional potential of bioactive compounds from kefir on blood pressure in physiological systems (Table 2).

*In situ*, the ACE activity was reduced by 98.4% in the milk after fermentation with kefir grains for 24 h. The effect observed was attributed to bioactive peptides released by kefir's microorganisms during the fermentation [14]. It suggests that bioactive peptides from kefir can have the potential to be used in the treatment of hypertension; however, further investigations are urgently needed to reach more consistent conclusions in the future.

#### 3.4.9. Antioxidant Effect in Aging Models Related to Neurodegenerative Diseases

Alzheimer's disease, Parkinson's disease, and amyotrophic lateral sclerosis are neurodegenerative diseases characterized by progressive loss of neuron cells, detriment of motor or cognitive functions, and accumulation of abnormal protein aggregates [105]. Aging is the primary risk factor for most neurodegenerative diseases, including Alzheimer's and Parkinson's disease. One in 10 individuals aged ≥65 has Alzheimer's disease, and the prevalence of the disease continues to increase as age advances [106]. Mitochondrial dysfunction and consequent oxidative stress during aging are well-established factors that significantly influence the progress of neurodegenerative diseases [106]. The reactive oxygen species (ROS) production in aged mitochondria increases, the membrane potential becomes smaller, the ATP synthesis reduces, and the activity of respiratory enzyme complexes declines.

Consequently, oxidation and aggregation of proteins occur, in addition to the oxidation of mitochondrial DNA, which starts to present mutations and deletions [105]. Few or no effective treatments for age-related neurodegenerative diseases are available, so they tend to progress irreversibly. In this context, stem cell therapy is one of the alternative methods studied for the treatment of neurodegenerative diseases to regenerate the neuronal population [107].

The present systematic review selected only two properly controlled studies testing bioactive compounds from kefir as a therapeutic strategy in neurodegenerative diseases. In both, the potential of EPS was assessed (Table 2). The effect of kefiran on the proliferation of neural stem cell culture (PC12 cell culture) was investigated by Jenab et al. [46] for 5 and 10% kefiran concentrations associated with pure poly-acrylonitrile (PAN) to form nanofibers. When comparing with the control (PAN alone), however, both kefiran concentrations tested reduced up to 26.7% of the PC12 viability after one-day incubation, showing no significant effect after two days. On the fourth and sixth days, 10% kefiran reduced by 15.4% and 21.2%, respectively, the viability of the PC12 cell line. Thus, kefiran was not a suitable compound for promoting neuronal regeneration; unlike, it showed toxicity from 10% concentration.

On the other hand, oral administration of EPS (20 mL EPS solution/kg bw) during 12 weeks had a beneficial effect on the aging mouse model induced with D-galactose since EPS could mitigate the resulting oxidative stress. At a low dose (1 mg/mL), EPS enhanced by 27.7% the total antioxidant capacity in the serum. However, in addition to increasing the total antioxidant capacity, EPS increased serum glutathione peroxidase, superoxide dismutase, and catalase by 21.55, 33.14, and 61.09%, respectively. A 49.6% reduction in serum malondialdehyde accompanied the increased activity of the antioxidant enzymes reported [83]. Malondialdehyde, in turn, has been proposed as a biological marker of the progression of neurodegenerative diseases, including Parkinson's disease, amyotrophic lateral sclerosis, and Alzheimer's disease. It is because the elevation of peripheral malondialdehyde levels occurs in these diseases [108]. Thus, the reduction of serum malondialdehyde suggests that EPS can mitigate the progression of neurodegenerative diseases.

*Lactobacillus plantarum* YW11 was identified as the EPS-producing microorganism with antioxidant activity (Table 2). The antioxidant effect of EPS at the peripheral level was attributed to its ability to modulate intestinal microbiota positively. This modulatory effect was associated with a reduction of oxidative stress in the intestinal tract; the decrease in NOx with a simultaneous increase in acetic and butyric acids in feces illustrates the potential of EPS in improving the oxidative conditions of the host's intestinal tract (Figure 2). Interestingly, EPS was more efficient than ascorbic acid in reducing the level of NOx and just as efficient in raising the content of short-chain fatty acids in the intestine. The antioxidant capacity of EPS can be attributed to its scavenging ability on hydroxyl radicals, DPPH radicals, and superoxide anion, in addition to Fe^2+^ chelating ability [83].

Therefore, EPSs from kefir seem more promising in terms of preventive effects than for therapeutic purposes. Indeed, the aging mouse model exhibits similar characteristics to those presented in the early stage of neurodegenerative diseases, such as oxidative stress. Therefore, this model is well-established for studying the earliest neurodegenerative changes associated with these diseases [109]. However, human clinical trials and more preclinical assays must be performed to corroborate this hypothesis.

#### 3.4.10. Effect on Osteoporosis

The systematic review here selected only 1 study on the effect of the bioactive compounds on osteoporosis [80]. However, the *in vivo* animal study did not investigate the microorganisms that produce the bioactive compound in kefir, nor its precursor in milk. The mechanisms of action have also not been described. Therefore, further studies are urgently needed better to understand the effect of kefir bioactive compounds on osteoporosis.

The ovariectomized procedure in female mice resulted in osteoporosis associated with the fall of the estrogen hormone due to the removal of the ovaries. These are the main glands that produce estrogen, which caused an imbalance between osteoblastic and osteoclastic activities in the bone. Consequently, there was a reduction of approximately 16%, 70%, and 70% of the bone mineral density, trabecular bone volume, and trabecular number in mice. However, the supplementation with bioactive peptides from kefir (100 mg/kg body weight) to ovariectomized mice could inhibit bone loss. Thus, the peptides increased the bone mineral density, trabecular bone volume, trabecular number, and the mechanical properties of the elastic modulus and hardness of the bones by 41%, 264%, 235%, 42%, and 36%, respectively. Consequently, a reduction of 36.5% and 33% in the trabecular separation and nanoindentation areas on the femur was obtained. It is important to note that peptide supplementation could ultimately reverse the condition of osteoporosis since the bone mineral density, trabecular number, trabecular separation, elastic modulus, hardness, and nanoindentation areas were similar to those of the negative control (nonovariectomized female).

Administration of calcium carbonate, standard procedure for osteoporosis treatment, also could enhance the trabecular bone volume and trabecular number. However, these values were about 0.7 times lower than those obtained by supplementation with bioactive peptides, demonstrating the potential of the peptides from kefir as a therapeutic agent in osteoporosis. Finally, no significant difference in the effects of calcium and bioactive peptides' combined action concerning isolated bioactive peptides revealed no synergism between these two treatments [80].

### 3.5. Do Bioactive Compounds from Artisanal and Industrial Kefir Differ in Terms of Their Functional Potential?

**Figure 3:**
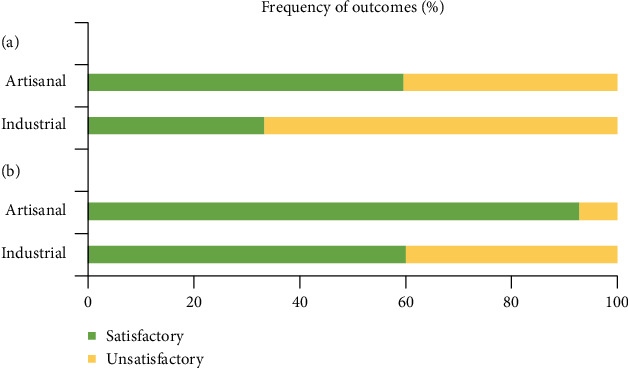
Frequency of significant satisfactory and unsatisfactory outcomes from artisanal and industrial kefir trials included in the meta-analysis. (a) Antimicrobial potential outcomes. (b) Antioxidant potential outcomes.

Two groups were assessed for comparing artisanal (kefir grain) and industrial (starter culture) kefir trials regarding their functional properties (Figure 3). First, we target effects related to antibacterial/antifungal potential resulting in an estimated OR of 2.96 (95% CI: 0.87–10.05, *P* = 0.07). Therefore, the type of kefir was not significantly associated with its antimicrobial potential. This result agrees with our findings (Table 2), which reported the organic acids (especially lactic acid) as one of the main responsible bioactive compounds for the antimicrobial effect in both types of kefir.

After aiming to evaluate artisanal and industrial kefir outcomes concerning antioxidant potential, another group analysis was carried out, resulting in an estimated OR of 8.56 (95% CI: 2.27–32.21, *P* ≤ .001). Therefore, the utilization of artisanal kefir may provide 756% higher chances of having antioxidant effects than kefir from the industrial process. As stated before, phenolic compounds were the bioactive compounds responsible for industrial kefir antioxidant activity. In contrast, artisanal kefir presented a greater diversity of bioactive antioxidant compounds; in addition to phenolic compounds, artisanal kefir also contained bioactive peptides and EPS, including kefiran (artisanal kefir-exclusive structure) (Table 2). Although the phenolic compounds in industrial kefir have reducing power, they were ineffective in terms of the radical scavenging ability [85], unlike the phenolics present in artisanal kefir [75]. These findings suggest a different profile of phenolic compounds between both kefirs that can affect the antioxidant potential. In addition, the greater diversity of bioactive compounds in artisanal kefir may have contributed to potentiate the antioxidant capacity of this type of kefir (Table 2).

The difference in the antioxidant potential may be related to the different microbial profiles between the types of kefir since the microorganisms reported as producers of the antioxidant compounds present in artisanal kefir [28, 73] are not commonly found in industrial kefir (Table 1). Indeed, artisanal kefir's more diverse microbial profile has been suggested as responsible for granting better functional benefits than industrial kefir [20]. Consistently, as discussed earlier, we observed a dramatically greater fungal diversity in artisanal kefir. In addition, the bacterial composition between both kefirs presented particularities (Table 1). Therefore, the microbial profile of artisanal kefir seems to be related to producing a greater diversity of bioactive compounds with antioxidant potential.

However, it is essential to report that because artisanal kefir is often primarily selected for kefir studies, only a few studies reporting industrial kefir findings were included in the meta-analysis, which was a limiting factor for comparison between kefir types.

## 4. Conclusion and Prospect

This review highlights the potential of bioactive compounds from kefir as preventive and therapeutic agents. There is an abundance of studies addressing the health benefits of kefir administration. However, a literature limitation is uncontrolled experiments or not identifying the bioactive compounds responsible for the observed health benefits. In addition, the relative scarcity of studies investigating the bioactive compounds-producer microorganisms and their substrates in milk constitutes a gap in the literature. The meta-analysis corroborated the antimicrobial, anticancer, and immune-modulatory activities. Kefiran and lactic acid were the main components with global antimicrobial action. However, kefiran presented a concentration threshold from which it exerted a bactericidal rather than a bacteriostatic effect. At the same time, lactic acid inhibited the germination of bacterial spores in a dose-dependent manner. Still, EPSs, including kefiran, were primarily responsible for the activity against colon and breast cancer. Nevertheless, the EPS concentration and intervention time were decisive for obtaining or not the anticancer effect, with EPS presenting a dose-dependent inhibitory effect for cervical carcinoma and colon cancer.

Regarding the anti-inflammatory effect, EPS, extracellular vesicles, and lactate were the main bioactive agents involved. However, EPS can also have an immunostimulatory effect, depending on its concentration and environmental conditions (absence or presence of inflammatory insult). To immunostimulate, the EPS concentration had to be dramatically higher, despite the intervention time similar to that used for the anti-inflammatory effect. A high dosage of extracellular vesicles seemed more effective against colitis than low dosages. Therefore, these factors must be carefully considered in future clinical applications. It is noteworthy that the studies were limited to *in vitro* and *in vivo* experiments, so clinical evidence is urgently needed to help advance the practical application of bioactive compounds from kefir. The mechanisms of action of bioactive compounds were diverse, indicating that they can act by different signaling pathways. Although antioxidant activity and gut modulation have shown a trend that favors kefir efficacy, they did not reach statistical significance, possibly due to the high heterogeneity of the data. Thus, standardization of the methodologies used to evaluate these effects will help compare different studies in the future. Bioactive compounds on plasma glucose level, neurodegenerative disease, lipid profile, blood pressure, and osteoporosis were inconclusive, and further studies are needed for consistent conclusions. Finally, artisanal and industrial kefir can have different functional potential depending on the health effect, which can be associated with differences in the microbial composition between both types of kefir.

## Figures and Tables

**Table 1:** Microbiological diversity in artisanal and industrial milk kefir.

Type of kefir	Microbial diversity	Source of kefir culture	References
Industrial	*A. syzygii* K03D05, *Lb. plantarum* K03D08	Chile	Dinamarca et al., 2021
	*Lb. plantarum* CIDCA 83114, *Kl. marxianus* CIDCA 8154, *Streptococcus thermophilus* CIDCA 321	Argentina	Kakisu et al., 2011
	*Kz. unispora*, *Kodamaea ohmeri*, *Sc. boulardii*, *Sc. cerevisiae*	Malaysia	Azhar et al., 2019
	*A. fabarum*, *A. orientalis*, *D. anomalus*, *Kl. marxianus*, *Kz. exígua*, *Kz. turicensis*, *Kz. unispora*, *Lb. kefiranofaciens ssp. kefiranofaciens*, *Lb. kefiranofaciens ssp. kefirgranum*, *Lb. kefiri*, *Lb. helveticus*, *Lb. paracasei*, *Lb. parakefiri*, *Lb. reutrei*, *Lc. lactis ssp. cremoris*, *Lc. lactis ssp. lactis*, *Ln. mesenteroides*, *Sc. cerevisiae*	Germany	Nejati et al., 2020
	*Lb. lactis*, *Lb. rhamnosus*, *Lb. plantarum*, *Lb. casei*, *Sc. florentinus*, *Ln. mesenteroides* subsp. *cremoris*, *Bif. lactis*, *Bif. longum*, *Bif. Breve*, *Lb. acidophilus*, *Lb. reuteri*, *Streptococcus diacetylactis*	Canada	Bourrie et al., 2021

Artisanal	*Aspergillus amstelodam*, *Cordyceps bassiana*, *Fusarium solani*, *Lb. casei*, *Lb. kefiranofaciens*, *Lb. kefiri*, *Lb. mali*, *Lb. paracasei*, *Lb. satsumensis*, *Lc. lactis*, *Lc. lactis ssp. cremoris*, *Lc. lactis ssp. lactis*, *Ln. mesenteroides*	Brazil	Brasiel et al., 2021 [37]; Leite et al., 2015 [32]; Vieira et al., 2017 [38]; Zanirati et al., 2015 [36]
	*Enterococcus durans*, *Lb. kefiri*, *Lc. lactis*, *Ln. mesenteroides* subsp. *dextranicum*	Taiwan	Chang-Liao et al., 2020
	*Lactobacillus* sp., *Lb. delbrueckii*, *Lb. kefiri*, *Lb. paracasei*, *Lb. plantarum*, *Lb. sakei*, *Lc. lactis*, *Ln. gelidum*, *Ln. mesenteroides*, *Pediococcus pentosaceus*	Russia	Khokhlacheva et al. 2015 [34]; Mantzourani et al., 2019 [39]
	*Cryptococcus* sp. *vega*, *Cyberlindnera jadinii*, *Davidiella tassiana*, *Dekkera bruxellensis*, *Dioszegia hungarica*, *Eurotium amstelodami*, *Ganoderma lucidum*, *Heterobasidion annosum*, *Kz. barnettii*, *Kz. Unispora*, *Kl. marxianus*, *Malassezia pachydermatis*, *Microdochium nivale*, *Naumovozyma Castelli*, *Penicillium* sp. *vega*, *Peziza campestres*, *Pichia fermentans*, *Pichia kudriavzevii*, *Sc. cerevisiae*, *Teratosphaeria knoxdaviesii*, *Wallemia sebi*, *Zygosaccharomyces lentus*	Ireland, United Kingdom, United States, Spain, France, Italy, Canada, Germany	Marsh et al., 2013 [33]
	*A. fabarum*, *A. okinawensis*, *A. orientalis*, *Enterococcus durans*, *Kz. unispora*, *Kl. marxianus*, *Lb. diolivorans*, *Lb. kefiri*, *Lb. kefirofaciens*, *Lb. otakiensis*, *Lb. paracasei*, *Lc. lactis*, *Sc. cerevisiae*	Turkey	Purutoglu et al., 2020
	*A. orleanensis*, *A. pasteurianus*, *Acidocella aluminiidurans*, *Gluconobacter morbifer*, *Lb. acidophilus*, *Lb. apis*, *Lb. casei*, *Lb. crispatus*, *Lb. delbrueckii*, *Lb. gigeriorum*, *Lb. helveticus*, *Lb. kefiranofaciens*, *Lb. paracasei*, *Lb. rhamnosus*, *Lb. ultunensis*, *Lc. lactis*, *Lent. Kefiri*, *Ln. mesenteroides*, *Streptococcus thermophilus*	South Korea, Ireland, Lithuania, Britain, the Caucuses	Sindi et al., 2020 [40]
	*Lactobacillus helveticus*	Indonesia	Raras et al. 2019

Artisanal and industrial	*A. syzygii*, *Alternaria tenuissima*, *Bacillus sporothermodurans*, *Cladosporium cladosporioides*, *Didymella negriana*, *Filobasidium magnus*, *Hanseniaspora thailandica*, *Kl. Marxianus*, *Kz. unispora*, *Lb. Kefiranofaciens*, *Lb. parakefiri*, *Lb. plantarum Lc. lactis*, *Ln. pseudomesenteroides*, *Sc cerevisiae*, *Pichia manshurica*, *Pichia orientalis*, *Pichia fermentans*, *Torulaspora delbrueckii Wickerhamiella pararugosa*	Bosnia and Herzegovina	Garofalo et al., 2020 [18]
	*A. lovaniensis*, *A. orientalis*, *Enterobacter amnigenus*, *Gluconobacter frateurii*, *Gluconobacter cerinus*, *Kz. khefir*, *Kl. marxianus*, *Lb. kefiranofaciens*, *Lb. parakefiri*, *Lb. kefiri*, *Lc. lactis*, *Ln. mesenteroids*, *Naumovozyma* sp.	Belgium	Korsak et al., 2015 [29]

*A.: Acetobacter*; *Bif.: Bifidobacterium*; *Kl.: Kluyveromyces*; *Kz.: Kazachstania*; *Lb.: Lactobacillus*; *Lc.: Lactococcus*; *Ln.: Leuconostoc*; *Sc.: Saccharomyces*.

**Table 2:** Effect and conditions of intervention with kefir bioactive compounds for different health benefits.

Health effect	Model/participants	Bioactive compound	MO^1^	Concentration tested	Intervention time	Effect tested/condition/bioactive compound concentration	Effect compared to control	Action mechanism	Reference
Anticarcinogenic	*In vitro*: MTT assay of MCF7 human breast cancer cell line	Kefiran	-	500 to 4000 *μ*g/mL	48 h	Cell viability		-	[46]
						500 to 2000 *μ*g/mL	↓up to 45%		
						4000 *μ*g/mL	Without effect		
					72 h	500 *μ*g/mL	Without effect		
						1000 to 4000 *μ*g/mL	↓up to 15.6%		
	*In vitro*: MTT assay of HT-29 human colon cancer cell line	Exopolysaccharide MSR101	*Lactobacillus kefiri* MSR101	50 to 400 *μ*g/mL	24 h	Cell viability	↓up to 55.9%	Upregulates the expression of apoptosis-related genes (Cyto-c, BAX, BAD, caspase 3, caspase 8, and caspase 9) in HT-29 cells	[47]
	*In vitro*: MTT assay of HepG2 human hepatocellular carcinoma cell line and HeLa human cervical carcinoma cell line	Kefiran	*Lactobacillus kefiranofaciens*	15.6 to 1000 *μ*g/mL	24 h	Cell viability HeLa cells	↓up to 72%	Adversely affected the morphological characteristics of HeLa and HepG2 carcinoma cell lines	[48]
						HepG2 cells			
						250 to 1000 *μ*g/mL	↓up to 82%		
						Below 250 *μ*g/mL	Without effect		
	*In vitro*: fecal water-induced DNA damage assay in HT-29 human colon cancer cell line	Lactic and acetic acids	-	20 to 200 *μ*L/mL	30 min (preincubation)	DNA damage		Antioxidant activity	[49]
						200 *μ*L/mL	↓ 20%		
						Below 200 *μ*L/mL	Without effect		
	*In vitro*: cell nuclei counting assay of MCF7-E3 human breast cancer estrogen-sensitive cells	Bioactive peptides	-	0.31 to 10% (v/v)	6 days	Cell number	↓up to 88%	-	[15]
Anti-inflammatory	*In vivo*: DSS-induced acute colitis model in male wild-type C57BL/6 mice; chronic colitis model aggravated by Piroxicam in IL10^−/−^ C57BL/6 mice	Extracellular vesicles (PRCC-1301 EVs)	*Lactobacillus kefirgranum* PRCC-1301	0.03 and 3 mg/kg bw/day	Acute colitis: from 2 days before administration of DSS to 5 days	Acute colitis		Promoting the intestinal barrier integrity through the expression of occlusion proteins (ZO-1, claudin-1, and occludin) in colon epithelial cells and inhibition of the NF-*κ*B signaling pathway in the distal and proximal colon in an acute colitis model	[50]
						Body weight	↑16%		
						Colon length (3 mg/kg bw)	↑29.6%		
						Histological score	↓up to 63%		
					Chronic colitis: from 14^th^ to 28^th^ day (Piroxicam from day 0 to day 14)	Chronic colitis			
						Body weight	Without effect		
						Colon length (3 mg/kg bw)	↑14.3%		
						Histological score	↓up to 85.3%		
	*In vitro*: RAW264.7 cell line (murine macrophages)	Exopolysaccharide R-17-EPS	*Lactobacillus pentosus* LZ-R-17	50 to 400 *μ*g/mL	24 h	Macrophage cell viability	↑up to 38%	-	[51]
						Phagocytosis index	↑up to 44%		
						Acid phosphatase activity	↑up to 78%		
						NO production	↑up to 97.2%		
						TNF-*α*			
						50 and 400 *μ*g/mL	Without effect		
						100 to 200 *μ*g/mL	↑up to 10.2%		
						IL-6			
						50 to 200 *μ*g/mL	↑up to 16.7%		
						400 *μ*g/mL	↓ 9.5%		
						IL-1*β*			
						50 and 400 *μ*g/mL	Without effect		
						100 to 200 *μ*g/mL	↑up to 14%		
						IL-10			
						100 to 400 *μ*g/mL	↑up to 6.4%		
						50 *μ*g/mL	↓ 6.4%		
	*In vitro*: DSS-induced acute colitis model in Caco-2 cell line cultures (human intestinal epithelial cells)	Extracellular vesicles	*Lactobacillus kefirgranum* PRCC-1301	100 *μ*g/mL	6 h	Cytokine gene expression:		-	Kang et al. (2020) [50]
						IL-2	↓58.3%		
						IL-8	↓64.3%		
						TNF-*α*	↓67%		
	*In vitro*: RAW264.7 cell line (murine macrophages)	Exopolysaccharide (R-5-EPS)	*Lactobacillus helveticus* LZ-R-5 from Tibetan kefir	50 to 400 *μ*g/mL	24 h	Macrophage cell viability	↑up to 19%	-	[52]
						Phagocytosis index	↑up to 35.9%		
						Acid phosphatase activity	↑up to 44%		
						NO production	↑up to 44.4%		
						TNF-*α*			
						50 *μ*g/mL	Without effect		
						100 to 200 *μ*g/mL	↑up to 25.5%		
						400 *μ*g/mL	↓27.5%		
						IL-6	↑up to 54.3%		
						IL-1*β*			
						100 and 200 *μ*g/mL	↑up to 20.5%		
						50 and 400 *μ*g/mL	↓up to 13.6%		
						IL-10			
						50 to 200 *μ*g/mL	↑up to 18.2%;		
						400 *μ*g/mL	Without effect		
	*In vitro*: PBMC culture isolated from human total peripheral blood	Kefiran	-	1000 and 5000 *μ*g/mL	48 h	IL-6	Control: not detected Treatment:	-	Jenab et al. (2020) [46]
						1000 *μ*g/mL	220 ng/L		
						5000 *μ*g/mL	270 ng/L		
				500 to 4000 *μ*g/mL	24 h, 48 h, 72 h, and 96 h	PBMC viability: 24 h			
						500 to 1000 *μ*g/mL	Without effect		
						2000 to 4000 *μ*g/mL	↑up to 200%		
						48 h to 96 h	Without effect		
	*In vitro*: TNF-*α*-induced intestinal inflammation model in Caco-2 cell line	Extracellular vesicles (80 to 400 nm)	*Lactobacillus kefir*, *Lactobacillus kefiranofaciens*, *Lactobacillus kefirgranum*	1 × 10^9^ extracellular vesicles/mL (A strains mix ratios: 1 : 1 : 1)	24 h	IL-8		Reducing the phosphorylation of p65, a subunit of NF-*κ*B	[53]
						mRNA level	↓up to 65.6%		
						Secretion	↓up to 96.8%		
	*In vitro*: a cell-free system containing sodium nitroprusside (10 mM)	Kefiran	-	5000 to 10.000 *μ*g/mL	2.5 h	NO radical production	↓up to 40.91%	Nitric oxide radical scavenging capacity of kefiran	[54]
	*In vitro*: FliC-induced intestinal inflammation model in Caco-2 cell line	Exopolysaccharide	*Lactobacillus paracasei* CIDCA 8339, CIDCA 83123, and CIDCA 83124 strains	*L. paracasei* suspensions (*OD*_590_ 0.25)	1 h (preincubation)	Promoter induction: CCL20	↓up to 55%	-	[13]
	*In vivo*: TNBS-induced inflammatory bowel disease in Balb/c mice	Extracellular vesicles	*Lactobacillus kefir*, *Lactobacillus kefiranofaciens*, and *Lactobacillus kefirgranum*	3 × 10^8^ and 3 × 10^10^ extracellular vesicles/head/day (A strain mix ratios: 1 : 1 : 1)	10 days	Body weight	↑up to 12.5%	Blocking MPO activation in mouse serum	Seo et al. (2018) [53]
						Rectal bleeding severity	↓up to 75%		
						Diarrheal conditions	↓up to 91.4%		
						Histopathological damage	↓up to 85%		
	*In vitro*: cell-free system	Polysaccharide extract	-	5000 *μ*g/mL	72 h	Hyaluronidase inhibition	↓up to 35%	-	[55]
	*In vitro*: intestinal inflammation model in Caco-2 cell line induced by IL-1*β*, TNF-*α*, or FliC	Lactate	-	100 mM	30 min (preincubation)	Promoter inhibition CCL20		Inhibition of the NF-*κ*B signaling pathway	[56]
						FliC-induced	↓78%		
						IL-1*β*-induced	↓80%		
						TNF-*α*-induced	↓42%		
	*In vitro*: peritoneal macrophages isolated from six-week Balb/c female mice	Protein (*molecular* *mass* > 30 kDa)	*Lactobacillus kefiranofaciens* M1	5 *μ*L of kefir supernatant/mL	24 h	Secretion		-	[57]
						TNF-*α*	↑1000%		
						IL-1*β*	↑700%		
						IL-6	↑1300%		
						IL-12	↑3000%		
	*In vivo*: Six- to 8-week-old BALB/c female mice	Kefiran	*Lactobacillus kefiranofaciens*	100 mg/kg bw/day	2, 5, or 7 days	Small intestine Mucosa		-	[58]
						IgA			
						IL-10	↑up to 50%		
						IL-6	↑up to 22%		
						IL-12	↑up to 33%		
						Fluid			
						IL-4	↑up to 164%		
						IL-12	↑up to 67.5%		
						Large intestine			
						IgA	↑up to 43%		
						IgG	↑up to 41.7%		
						IL-4	↑up to 44.4%		
						IL-10	↑up to 47.2%		
						IL-6	↑up to 30%		
						IFN	↑up to 21.2%		
						TNF	↑up to 20%		
						Serum:			
						IL-4	↑ up to 209%		
						IL-6	↑ up to 254%		
						IL-10	↑ up to 74.5%		
						IFN	↑ up to 170%		
	*In vivo*: cotton-induced granuloma in Wistar rats	Kefiran	-	1 mL kefir suspension/day	7 days	Granuloma weight	↓44%	-	[16]
[59]	*In vitro*: *Klebsiella pneumoniae* (KP), *Pseudomonas aeruginosa* (PA), *Bacillus cereus* (BC), *Staphylococcus aureus* (SA)*, Staphylococcus epidermidis* (SE), *Escherichia coli* (EC), clinical isolates *Proteus mirabilis* (PM), and *Listeria monocytogenes* (LM)	Lactic acid	Cow milk kefir: mesophilic aerobic bacteria, yeast, *Lactobacillus*, and *Lactoccoccus*	Cow milk kefir: 0.90% (w/w)	24 h	Microorganism growth Cow's milk kefir		-	[60]
						BC	↓132.33%		
						KP	↓72.05%		
						SA	↓33.33%		
						SE	↓31.89%		
						LM	↓16.66%		
						PA	Without effect		
						EC	Without effect		
						PM	Without effect		
			Donkey milk kefir: mesophilic aerobic bacteria, yeast, *Lactobacillus*, *Lactoccoccus*, and *Leukonostoc*	Donkey milk kefir: 0.80% (w/w)		Donkey milk kefir			
						BC	↓183.33%		
						KP	↓17.39%		
						SA	↓4.34%		
						SE	↓16.89%		
						LM	↓58.33%		
						PA	Without effect		
						EC	↓33.33%		
						PM	↓58.33%		
	*In vitro*: *Escherichia coli* (EC), *Salmonella* Typhimurium (ST), and *Staphylococcus aureus* (SA)	Lactic, acetic, and pyruvic acids	*Acetobacter orientalis*, *Lactococcus lactis*, *Lactobacillus gallinarum*, *Kazachstania unispora*, *Pichia kudriavzevii*, *Galactomyces candidum*, *Geotrichum bryndzae*, *Lactobacillus kefiri*, and *Saccharomyces cerevisiae*	25, 50, 75, and 100% (v/v)	48 h	Microorganism growth		-	[14]
						EC	↓100%		
						ST	↓100%		
						SA	↓98.6% to 100%		
	*In vitro*: *Staphylococcus aureus* (SA), *Streptococcus faecalis* (SF), *Pseudomonas aeruginosa* (PA), and *Escherichia coli* (EC)	Kefiran	-	1% (w/v)	24 h	Zone of inhibition Kefiran extracted by hot water			[61]
						EC	↓41.6%		
						PA	↓51.3%		
						SF	↓60.2%		
						AS	↓61.6%		
						Kefiran extracted by ultrasound			
						EC	↓33.7%		
						PA	↓43.0%		
						SF	↓50.4%		
						AS	↓51.4%		
						Kefiran (hot water+ultrasound)			
						EC	↓23.6%		
						PA	↓32.8%		
						SF	↓42.9%		
						AS	↓43.4%		
	*In vitro*: *Pseudomonas aeruginosa* (PA) and methicillin-resistant *Staphylococcus aureus* (MRSA)	FK-1000 (composed of sugars and amino acids)	*Lactobacilacea*, *Acetobacteraceae*, *Pseudomonadacea*, *Streptococcaceae*, *Leuconostocaceae*, *Enterobacteriaceae*, *Alphaproteobacteria*, *Aeromonadaceae*, and *Pseudomonadales*	200 *μ*L/well of Muller Hinton	18 h	Microorganism growth pH 5		-	[62]
						MRSA	↓100%,		
						PA	↓100%		
						pH 7			
						MRSA	↓83%		
						PA	Without effect		
	*In vitro*: *Pseudomonas putida* extracted from spoiled chicken	Fraction with *MM* < 6000 in the supernatant	*Lactobacillus paracasei FX-6*	0.078%, 0.156%, 0.3125%, 0.625%, 1.25%, 2.5%, 5.0%, and 10% (w/v)	21 h	Microorganism growth *Pseudomonas putida* 1.25% (w/v)	↓13.5% to 98%	Performance on the plasma membrane, DNA and proteins of pathogenic microorganisms	[63]
	*In vitro*: *Escherichia coli* (EC), *Lactobacillus plantarum* (LP), *Micrococcus luteus* (ML), *Listeria monocytogenes* (LM), *Salmonella enteritidis* (SE), *Staphylococcus aureus* (AS), *and Bacillus cereus* (BC)	Bacteriocin	-	100, 150, 200, and 250 *μ*L/wells with a diameter of 7 mm	24 h	Zone of inhibition		-	[40]
						LP	↑up to 15 times		
						ML	↑up to 20 times		
						BC	↑up to 7 times		
						LM	↑up to 14 times		
						SA	↑up to 8 times		
						SE	↑up to 10 times		
						EC	Without effect		
	*In vitro*: *Escherichia coli* (EC), *Klebsiella pneumoniae* (KP), *Pseudomonas aeruginosa* (PA), *Enterococcus faecalis* (EF), *Bacillus cereus* (BC)*, Bacillus subtilis* (BS), *and Staphylococcus aureus* (SA)	Bioactive peptides	*Lactobacillus*, *Lactococcus*, and yeast	2.5% (w/v)	24 h	Antimicrobial activity		-	[64]
						SA	↑up to 100%		
						EC	↑80% to 100%		
						KP	↑up to 99.98%		
						EF	↑94.5%to100%		
						BS	↑up to 75.2%;		
						PA	↑34.7% to 51%		
						BC	↑up to 12.2%		
	*In vitro*: *Listeria monocytogenes* (LM) and *Salmonella* enteritidis (SE)	Exopolysaccharide DN1	*Lactobacillus kefiranofaciens* DN1	0.35, 1% and 2.5% (w/v)	24 h	Microorganism growth 0.3% (w/v)		-	[65]
						LM	↓56%		
						SE	↓5.45%		
						1% and 2.5% (w/v)			
						LM	↓100%		
						SE	↓100%		
	*In vitro*: *Escherichia coli* (EC), *Bacillus cereus* (BC), and *Salmonella* enteritidis (SE)	Lactic Acid	-	25, 50, 75, and 100% (v/v)	24 h	Microorganism growth 50-100% (v/v)		-	[66]
						EC	↓31% to 99%		
						SE	↓up to 98.7%		
						BC	↓up to 87%		
						25% (v/v)			
						SE	↑6%		
						BC	↑24%		
	In vitro: methicillin-resistant *Staphylococcus aureus* (MRSA) (S547)	Biofilms	*Lactobacillus plantarum*	10^6^ CFU/mL	12, 24, 36, 48, 60, 72, and 84 h	Microorganism growth MRSA	↓1.4% to 30%	Inhibition of biofilm production by MRSA	[67]
	*In vitro*: *Escherichia coli* (EC)	F1 bioactive peptide	*Lactobacillus paracasei* subsp. *Tolerans FX-6*	0.00625% (w/v)	20 h	Microorganism growth EC	↓33% to 57%	Damage to the outer and inner cell membrane with extravasation of potassium ions and cytoplasmic *β*-galactosidase; binding to the bacterial DNA	[68]
	*In vitro*: *Escherichia coli* (EC), *Klebsiella pneumoniae* (KP), *Pseudomonas aeruginosa* (PA), *Salmonella* Typhymurium (ST), and *Staphylococcus aureus* (SA)	Kefiran	*Lactobacillus kefiranofaciens*	1% (w/v)	13 h	Microorganism growth		-	[69]
						EC	↓up to 25.2%		
						PA	↓up to 19%		
						KP	↓up to 9.2%		
						ST	↓up to 3.3%		
						AS	↓up to 2%		
	*In situ* (whey fermented with kefir grains): *Aspergillus flavus* (AFL), *Penicillium crustosum* (PC), *Aspergillus terreus* (AT), *Aspergillus* *Fumigatus* (AF), *Trichoderma longibrachiatum* (TL), *Rhizopus sp.*, and *Aspergillus parasiticus* (AP)	Lactic and acetic acids	-	95% (v/v)	24 h	Microorganism germination		-	[70]
						*Rhizopus* sp.	↓70%		
						AP	↓67		
						TL	↓61%		
						AF	↓60%		
						AFL	↓34%		
						PC	Without effect		
						AT	Without effect		
	*In vitro*: spent culture supernatant (SCS) obtained from *Clostridium difficile*	*Thermolabile* *fraction* > 10 kDa in the supernatant	-	*OD*_550_ = 1	1 h	Biological activity of *C. difficile* SCS on Vero cells Supernatant from *Lactococcus lactis* subps. *lactis* or from a mixture of all microorganisms of kefir	↓80%	-	
						Supernatant from *Lactobacillus kefir*, *Lb. plantarum*, *Saccharomyces cerevisiae*, and *Kluyveromyces marxianus*	Without effect		
	*In vitro*: *Streptococcus faecalis* (SF), *Pseudomonas aeruginosa* (PA), *Salmonella* Typhi (STP), *Bacillus subtilis* (BS), *Bacillus cereus* (BC), *Escherichia coli* (EC), *Klebsiella pneumoniae* (KP), *Staphylococcus aureus* (SA), and *Fusarium graminearum* (FG) *Aspergillus flavus* AH3 (mycelial dry weights and aflatoxin B1)	Kefiran	-	0.1 mL/5 mm diameter paper disks 0.5, 1.0, 2.0, 3.0, 4.0, 5.0, 6.0, 7.0, 8.0, 9.0, and 10% (v/v)	24 h for bacteria and yeasts and 7 days for fungi 10 days	Zone of inhibition		-	[71]
						STP	↑75%		
						SF	↑66.6%		
						BC	↑62.5%		
						PA	↑50%		
						BS	↑44.4%		
						EC	↑37.5%		
						SA	↑16.6%		
						FG	↑15.4%		
						KP	↑9.09%		
						Aflatoxin B1	↓33.3% to 100%		
						Mycelial dry weights	↓14.9% to 100%		
	*In vitro*: *Salmonella Typhimurium* (ST), *Escherichia coli* (EC), *Pseudomonas aeruginosa* (PA), and *Staphylococcus aureus* (SA)	Lactic acid (partial effect)	-	0.9% (w/w)	24 h	Zone of inhibition		-	[17] – Industrial kefir
						ST	↓60%		
						SA	↓56.6%		
						EC	↓56.5%		
						PA	Without effect		
					48 h	ST	↓41.4%		
						SA	↓42.1%		
						EC	Without effect		
						PA	Without effect		
					7 days	ST	↓1.67%		
						SA	Without effect		
						EC	Without effect		
						PA	Without effect		
	*In vitro*: *Salmonella* enteritidis (SE)	S-layer proteins	*Lactobacillus kefir* strains CIDCA 8344 and CIDCA 8348	2 × 10^8^ CFU/mL	4 h	Microorganism growth S-layer proteins from *L. kefir* 8348		-	[72]
						*Salmonella* enteritidis	↓up to 99.96%		
						S-layer proteins from *L. kefir* 8344			
						*Salmonella* enteritidis	↓50% to 99.93%		
	*In situ* (kefir-fermented milk): spores and vegetative cells de *Bacillus cereus* and toxin production by *B. cereus*	Organic acids	-	1% and 5% (w/v)	24 h	1% (w/v)		Reduction of the biological activity of the pathogenic microorganism	[31]
						Vegetative cells	↓70% to 98%		
						Number of spores	↓up to 80 times		
						5% (w/v)			
						Vegetative cells	↓70% to 99.8%		
						Number of spores	↓from 80 to 50,000 times		
Antioxidant	*In vitro*: 2,2-diphenyl-1-picrylhydrazyl—DPPH; reduce Fe^3+^ to Fe^2+^ (FRAP)	Kefiran	-	0.08%, 0.04%, 0.02%, 0.01%, and 0.005% (w/v)	30 min	DPPH	↓25 to 85%	-	[61]
						FRAP	↓37 to 84%		
	*In situ* (cow milk kefir): 2,2′-azino-bis-3-ethylbenzothiazoline-6-sulfonic acid—ABTS; oxygen radical absorbance capacity—ORAC	Bioactive peptides	-	-	-	ABTS	↑35.8%	The antioxidant effect cannot be attributed to the ion chelating ability	[14]
				20 *μ*L/well (v/v)	80 min	ORAC	↑111.6%		
	*In vitro*: ascorbic acid equivalent reducing capacity (AAEC)	Kefiran	-	1% and 0.5% (w/v)	20 min	Ascorbic acid equivalent reducing capacity	Control (hyaluronic acid) Without effect Treatment	Metal chelating activity and sequestering activity of hydroxyl and superoxide radical	[54]
						0.5% (w/v)	4.44 *μ*g/mL		
						1% (w/v)	8.47 *μ*g/mL		
	*In situ* (cow and ewe milk kefirs) 2,2′-Azino-bis-3-ethylbenzothiazoline-6-sulfonic acid—ABTS 2,2-Diphenyl-1-picrylhydrazyl—DPPH Reduce Fe^3+^ to Fe^2+^ (FRAP)	Total phenolic compounds	-	5% (v/v) 8.3% (v/v) 8.3% (v/v)	3 min 30 min 30 min	Ewe milk kefir		-	[73, 74] – Industrial kefir
						ABTS	↓4.6% to 46%		
						DPPH	↓20% to 50%		
						FRAP	↑24% to 134%		
						Cow milk kefir			
						ABTS	↓52% to 70%		
						DPPH	↑70% to 220%		
						FRAP	↑13% to 120%		
	*In vitro* 2,2′-Azino-bis-3-ethylbenzothiazoline-6-sulfonic acid–ABTS 2,2-Diphenyl-1-picrylhydrazyl–DPPH	Bioactive peptides	-	0.625% (w/v)	6, 30, 60, 90, 150, and 180 min	ABTS	↑2% to 25.6%	-	[64]
				2.5%, 1.25%, 0.62, and 0.31% (w/v)	120 min	DPPH			
						2.5% peptides 1.25% peptides	↓2.64% to 17.7%		
						7 days	↑2.99%		
						14 days	↓0.3% to 6.32%		
						0.62% peptides			
						7 days	↓1.15% to 11.03%		
						From 14 days	↑13.22% to 18.85%		
						0.31% peptides	↓17.0% to 30.0%		
	In vitro: 2,2-diphenyl-1-picrylhydrazyl—DPPH	Exopolysaccharides	*Bacillus amyloliquefaciens*, *Uncultured Bacillus* sp*. clone*, *Bacillus subtilis*, *Bacillus subtilis*, *Bacillus tequilensis*, and *↓Bacillus siamensis*	1 mL (10^9^ CFU/mL)/3 mL	30 min	Antioxidant activity	↑10% to 20%	Resistance to hydrogen peroxide	[73]
	*In vitro*: 2,20-azobis(2-methypropionamidine) dihydrochloride)—APPH—in bovine serum albumin (BSA)	Exopolysaccharides	*Acetobacter okinawensis*, *Leuconostoc pseudomesenteroides*, and *Kazachstania unispora*	0.05%, 0.1%, 0.15%, 0.20%, and 0.25% (w/v)	360 min	AAPH-oxidized BSA protein	↓31% to 96%	-	[28]
	*In situ* (cow milk kefir): 2,2′-azino-bis-3-ethylbenzothiazoline-6-sulfonic acid—ABTS	Exopolysaccharides	-	0.00113% (w/v)	6 min	Antioxidant activity	↑8.43%	-	[74]
	*In situ* (goat milk kefir): 2,2′-azino-bis-3-ethylbenzothiazoline-6-sulfonic acid—ABTS; oxygen radical absorbance capacity—ORAC	Phenolic compounds	-	38.93, 61.60, 26.62, and 123.23% (w/v)	-	ABTS	↑62% to 120%	-	[75]
						ORAC	↑10% to 40%		
Cholesterol	*In-vitro*: exopolysaccharides (EPSs) in DMEM medium containing 3T3-L1 adipocytes	Exopolysaccharides	*Lactobacillus kefiri* (LKDH1, LKDH3, and LKDH5 strains) and *Leuconostoc mesenteroides* (LMDH4, LMDH6, LMDH7, LMDH8, and LMDH9 strains)	0.01 mg/mL, 0.1 mg/mL, and 0.2 mg/mL of exopolysaccharides isolated from their producer microorganisms	6 days	Lipid accumulation in adipocytes		-	[76]
						0.01 mg/dL LMDH4	↓12%		
						Other strains	Without effect		
						0.1 mg/dL			
						LMDH4	↓22%		
						LMDH7	↓24%		
						LKDH5	↓14%		
						Other strains	Without effect		
						0.2 mg/dL			
						LMDH7	↓28%		
						LDMH4	↓22%		
						LKDH3	↓22%		
						LKDH5	↓19%		
						LMDH6	↓10%		
						Other strains	Without effect		
	*In vivo*: C57BL/6J high-fat and high-fructose diet-induced obese mice and with oral administration of heat-killed lactic acid bacteria (HLAB) from kefir	Exopolysaccharides	*Leuconostoc mesenteroides* LMDH4 and *Lactobacillus kefiri* LKDH5	10 mL/kg bw of a mixture L*euconostoc mesenteroides* LMDH4 (1 × 10^10^ CFU/mL) and *Lactobacillus kefiri* LKDH5 (1 × 10^9^ CFU/mL)	8 weeks	Total cholesterol	Without effect	Reduction in the proinflammatory genes expression (*Hp* and *Wfdc21*) and genes (*Fabp4* and *Fasn*) related to the synthesis of fatty acids in the adipose tissues	[76]
						HDL	Without effect		
						LDL	Without effect		
						Triglyceride	Without effect		
						Adipose tissue weight	↓36%		
	*In vitro*: *Lactococcus lactis WH-C1* from kefir in GM17 broth supplemented with cholesterol (30 mg/100 mL)	Exopolysaccharides	*Lactococcus lactis* WH-C1	4% (v/v) inoculum *Lactococcus lactis WH-C1* from Tibet kefir grains	24 h	Cholesterol in the medium	↓up to 31.23%	-	[77]
Blood pressure	*In situ* (cow milk kefir): assay of inhibitory activity on angiotensin-converting enzyme (ACE)	Bioactive peptides	-	11.2 mg/100 mL	24 h	ACE activity	↓98.4%	-	[14]
Glucose	*In vivo*: C57BL/6J high-fat and high-fructose diet-induced obese mice and with oral administration of heat-killed lactic acid bacteria (HLAB) from kefir	Exopolysaccharides	*Leuconostoc mesenteroides* LMDH4 and *Lactobacillus kefiri* LKDH5	10 mL/kg bw of a mixture *Leuconostoc mesenteroides* LMDH4 (1 × 10^10^ CFU/mL) and *Lactobacillus kefiri* LKDH5 (1 × 10^9^ CFU/mL)	8 weeks	Plasma glucose	Without effect	-	[76]
	*In vivo*: Streptozotocin-nicotinamide- (STZ-NA-) induced diabetic rats/male rats of 8-12 weeks old	Alcohol and phenolic compounds	-	5 to 20 mL kefir with black rice extract (1 : 1)/kg bw	4 weeks	Number of Langerhans islet	↑up to 199%	Proton-radical scavenging activity	[78]
						Insulin-positive *β*-cells	↑up to 2330%		
Intestinal microbiota modulation	*In vitro*: fecal samples from healthy children aged between 8 months and 3 years old	Exopolysaccharides 8339 and 83124	*L. paracasei* CIDCA 8339 and CIDCA 83124	0.3% (w/v)	72 h	*L. paracasei* CIDCA 8339		-	[79]
						*Lentisphaerae*	↑32%		
						*Firmicutes*	↑12%		
						*Victivallis*	↑33%		
						*Acidaminococcu*	↑15%		
						*Comamonas*	*↑*6%		
						*Proteobacteria*	↓31%		
						*Bacteroidetes*	↓11%		
						*Actinobacteria*	↓1.5%		
						*L. paracasei* CIDCA 83124			
						*Proteobacteria*	↑15%		
						*Comamonas*	↑52%		
						*Firmicutes*	↓17%		
						*Bacteroidetes*	↓10%		
						*Actinobacteria*	↓1.3%		
						*Escherichia*	↓28%		
						*Bacteroides*	↓11% to 12%		
						*Klebsiella*	↓6%		
	*In vivo*: thirty 16-week-old female C57BL/6J ovariectomized mice	Bioactive peptides	-	100 mg of bioactive peptides/kg bw	56 days	*Firmicutes*/*Bacteroidetes*	↑17%	-	[80]
						*Alloprevotella*	↑326%		
						*Romboustsia*	↓85%		
						*Anaerostipes*	↓66%		
						*Ruminococcus*	↓55%		
						*Parasutterella*	↓46%		
						*Streptococcus*	↓39%		
	*In vivo*: male Balb/c mice aged 16 weeks	Exopolysaccharide	*Lactobacillus kefiranofaciens* XL10	0.4 mL of an XL10 suspension (10^8^ CFU/mL)	21 days	*Firmicutes/Bacteroidetes*	↑0.04% to 1.8%	-	[81]
						*Lactobacillaceae*	↑14.59%		
						*Ruminococcaceae* (day 7)	↑15.12%		
						*Bifidobacteriaceae*	↑0.2% to 0.59%		
						*Rikenellaceae*	↓2.63% to 2.74%		
						Abundance in the gender	↓ up to 39.19%		
	BALB/c female mice (6 to 8 weeks old)	Kefiran	-	Daily intake 0.75 to 1 mg kefiran per day	21 days	*Bifidobacteria*	↑ up to 17%	-	[82]
	*In vitro*: *Bifidobacterium bifidum* PRL2010	Kefiran	-	0.3% (w/v)	72 h	*Bifidobacterium bifidum* PRL2010	Control (MRSc without carbon source) Without growth Treatment 5.8 × 10^8^ CFU/mL	Enhanced transcription of genes that act as effector molecules in the microbe-host interaction, such as pili; transcription of genes involved in the metabolism of diet glucans	[41]
Nervous system	*In vitro*: MTT assay of PC12 cell line (from rat adrenal gland -phaeochromocytoma)	Kefiran	-	5 and 10%	1 to 6 days	Cell viability		-	[46]
						1 day	↓up to 26.7%		
						2 days	Without effect		
						4 days (10%)	↓15.4%		
						6 days (10%)	↓21.2%		
	*In vivo*: aging mouse model induced with D-galactose (oxidative stress)	Exopolysaccharide	*Lactobacillus plantarum* YW11	Low dose: 20 mL/kg bw/day of 1 mg/mL EPS solution High dose: 20 mL/kg bw/day of 2.5 mg/mL EPS solution	12 weeks	Low dose		Modulation of gut microbiota and reduction of oxidative stress of the intestinal tract (decrease of NOx fecal content and increase of content of short-chain fatty acids—acetic and butyric)	[83]
						T-AOC	↑27.7%		
						MDA	Without effect		
						GSH-Px	Without effect		
						SOD	Without effect		
						CAT	Without effect		
						High dose			
						T-AOC	↑38.18%		
						MDA	↓49.6%		
						GSH-Px	↑21.55%		
						SOD	↑33.14%		
						CAT	↑61.09%		
Osteoporosis	*In vivo*: thirty 16-week-old female C57BL/6J ovariectomized mice	Bioactive peptides (KPS)	-	100 mg of KPs/kg bw	56 days	Trabecular bone volume	↑264%	-	[80]
						Trabecular number	↑235%		
						Bone mineral density	↑41%		
						Mechanical properties	↑42%		
						Hardness of the bones	↑36%		
						Trabecular separation areas	↓36.5%		
						Nanoindentation areas	↓33%		

^1^Producer microorganism. ↑: increase; ↓: decrease; EPS: exopolysaccharide; IL: interleukin; TNF-*α*: tumor necrosis factor-*α*; IFN: interferon; ZO-1: zonula occludens-1; NO: nitric oxide; PBMC: peripheral blood mononuclear cell; DSS: dextran sulfate sodium; TNBS: 2,4,6-trinitrobenzene sulfonic acid; FliC: flagellin; DO: optical density; CCL20: chemokine-ligand-20; bw: body weight; MPO: myeloperoxidase; MDA: malondialdehyde; GSH-Px: glutathione peroxidase; SOD: superoxide dismutase; CAT: catalase; T-AOC: total antioxidant capacity; MTT: (3-(4,5-dimethylthiazol-2-yl)-2,5-diphenyl); Cyto-c: cytochrome c; BAX: BCL2-associated X; BAD: BCL2-associated agonist of cell death.

## Data Availability

The data used to support the findings of this study are included within the article.

## Supplementary Materials

Supplementary Materials Figure S1: forest plot of the standardized mean difference (SMD) and 95% confidence intervals for studies concerning the presence or lack of antimicrobial effect of kefir bioactive compounds compared to control treatments; Figure S2: forest plot of the standardized mean difference (SMD) and 95% confidence intervals for studies concerning the presence or lack of antioxidant effect of kefir bioactive compounds compared to control treatments; Figure S3: forest plot of the standardized mean difference (SMD) and 95% confidence intervals for studies concerning the presence or lack of gut microbiota modulation effect of kefir bioactive compounds compared to control treatments; Figure S4: forest plot of the standardized mean difference (SMD) and 95% confidence intervals for studies concerning the presence or lack of immune modulation effect of kefir bioactive compounds compared to control treatments; Figure S5: forest plot of the standardized mean difference (SMD) and 95% confidence intervals for studies concerning the presence or lack of anticancer effect of kefir bioactive compounds compared to control treatments.
