# Supplementary material for: Bioactive Compounds from Kefir and Their Potential Benefits on Health: A Systematic Review and Meta-Analysis
Source: Oxid Med Cell Longev. 2021 Oct 27;2021:9081738. doi: 10.1155/2021/9081738 (PMC8566050; doi:10.1155/2021/9081738)
Supplement: Supplementary Materials — Figure S1: forest plot of the standardized mean difference (SMD) and 95% confidence intervals for studies concerning the presence or lack of antimicrobial effect of kefir bioactive compounds compared to control treatments; Figure S2: forest plot of the standardized mean difference (SMD) and 95% confidence intervals for studies concerning the presence or lack of antioxidant effect of kefir bioactive compounds compared to control treatments; Figure S3: forest plot of the standardized mean difference (SMD) and 95% confidence intervals for studies concerning the presence or lack of gut microbiota modulation effect of kefir bioactive compounds compared to control treatments; Figure S4: forest plot of the standardized mean difference (SMD) and 95% confidence intervals for studies concerning the presence or lack of immune modulation effect of kefir bioactive compounds compared to control treatments; Figure S5: forest plot of the standardized mean difference (SMD) and 95% confidence intervals for studies concerning the presence or lack of anticancer effect of kefir bioactive compounds compared to control treatments. [file 9081738.f1.docx]

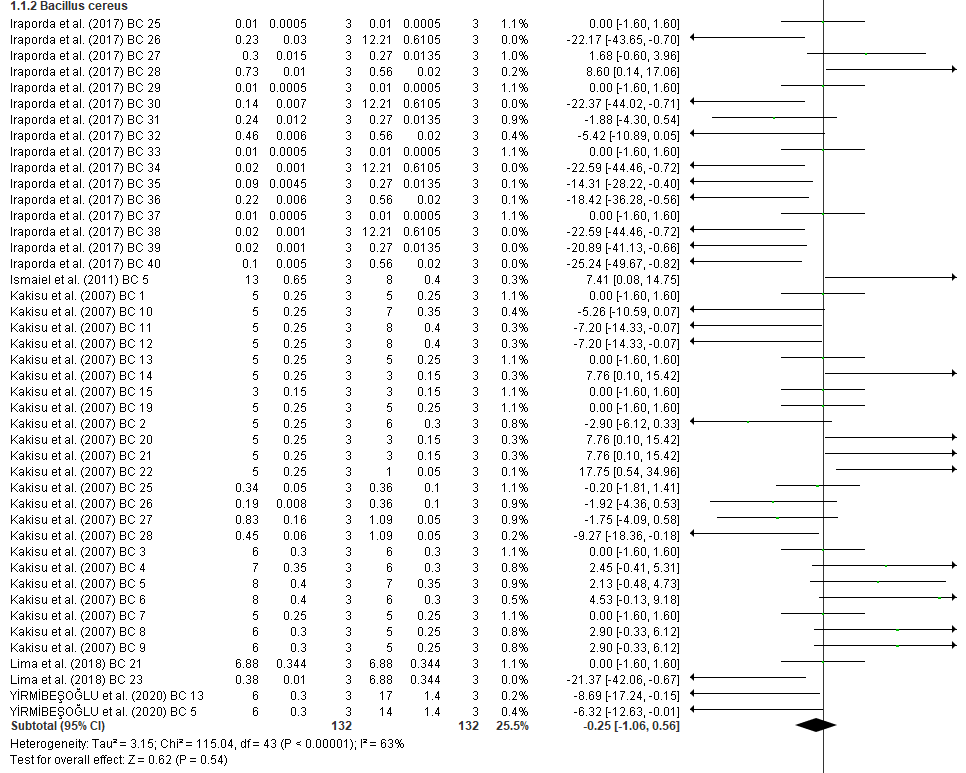

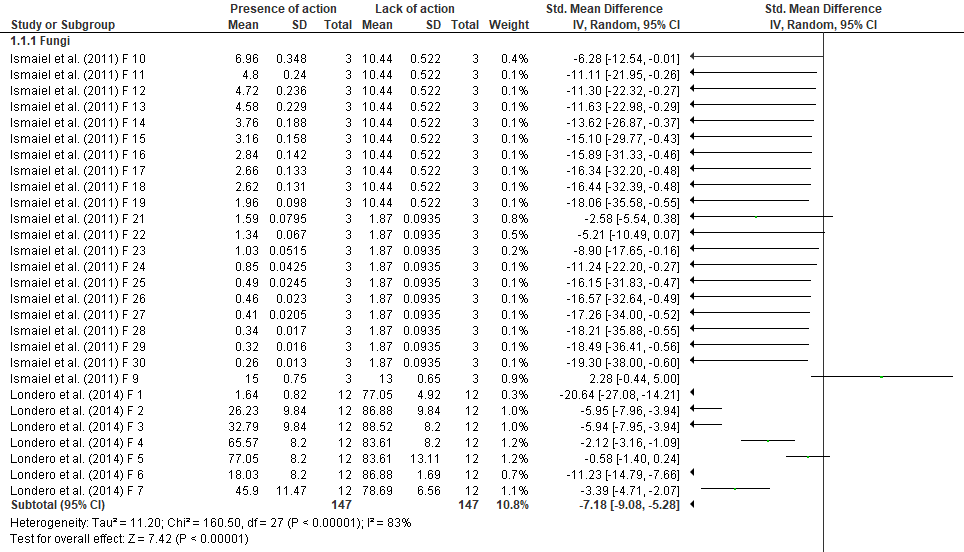


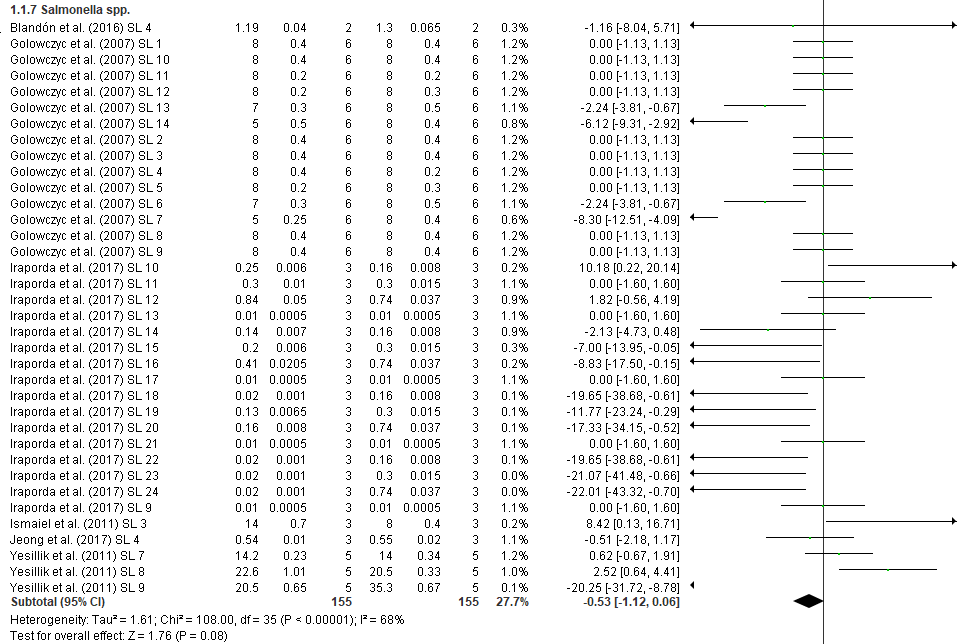

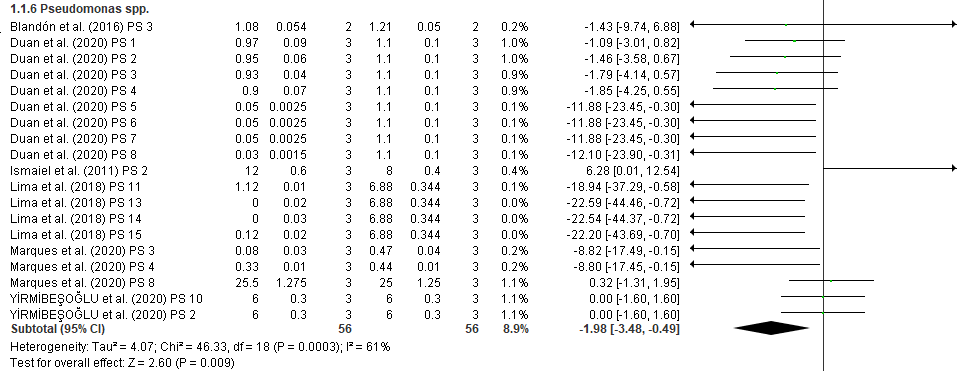

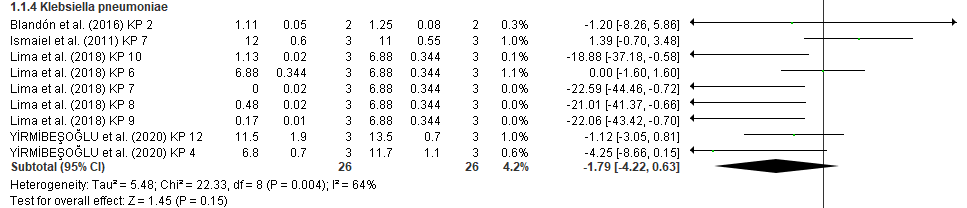

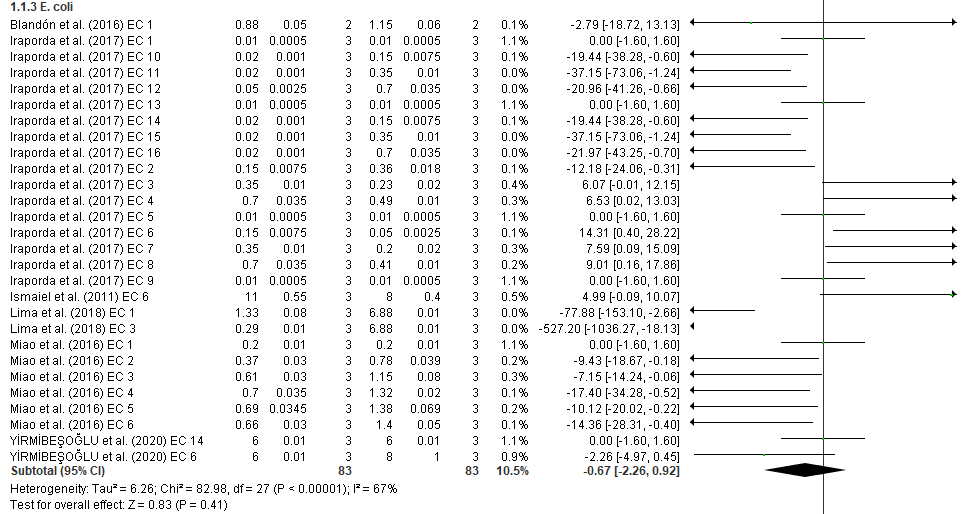


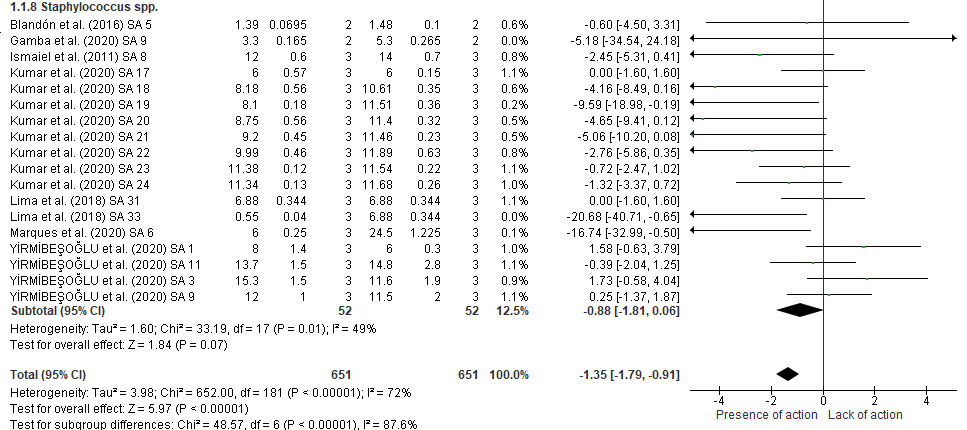


**Supplementary Figure S1.** Forest plot of the standardized mean difference (SMD) and 95% confidence intervals for studies concerning the presence or lack of antimicrobial effect of kefir bioactive compounds compared to control treatments. Each line contains the point outcomes and confidence intervals. Green boxes represent the relative weight of each study. Combined effects estimates are represented by a diamond symbol at the bottom of each category.


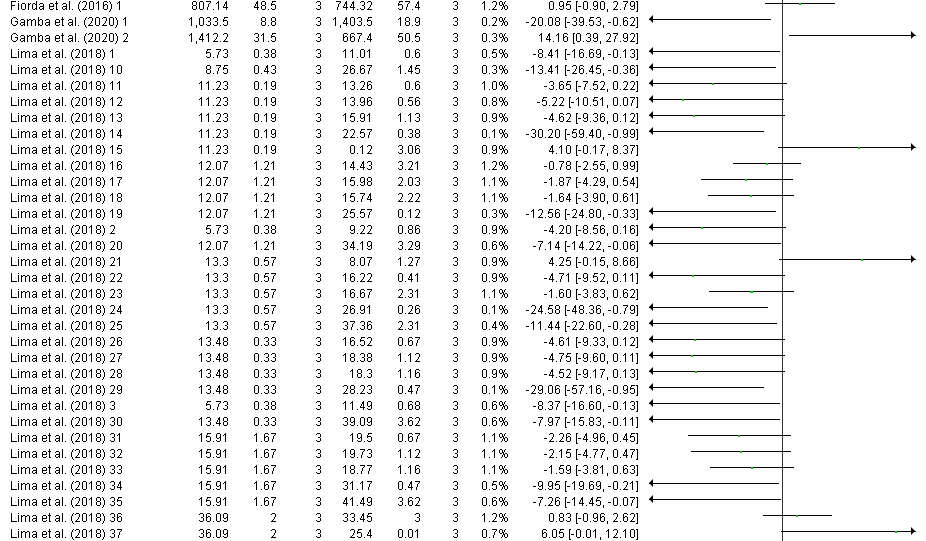

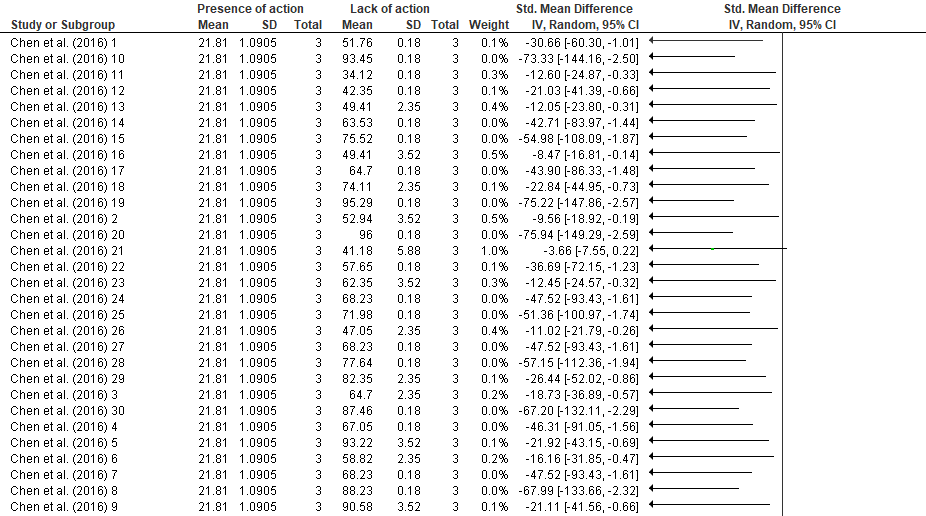

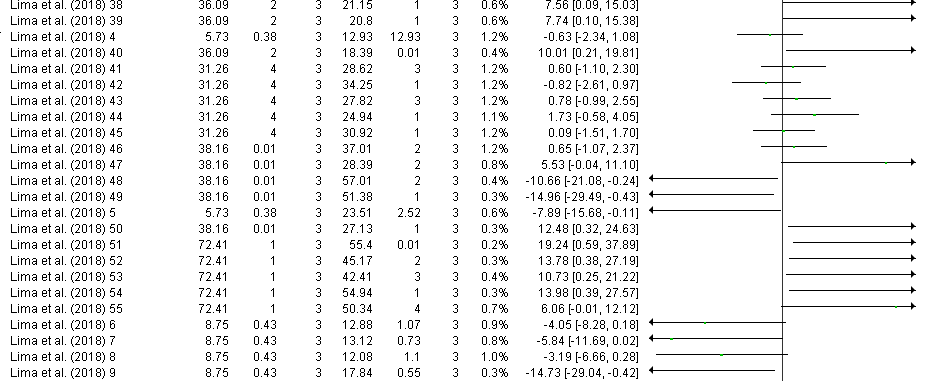


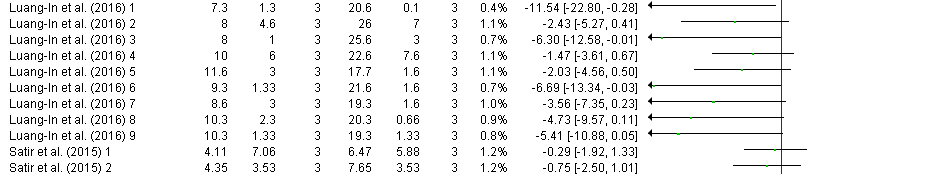

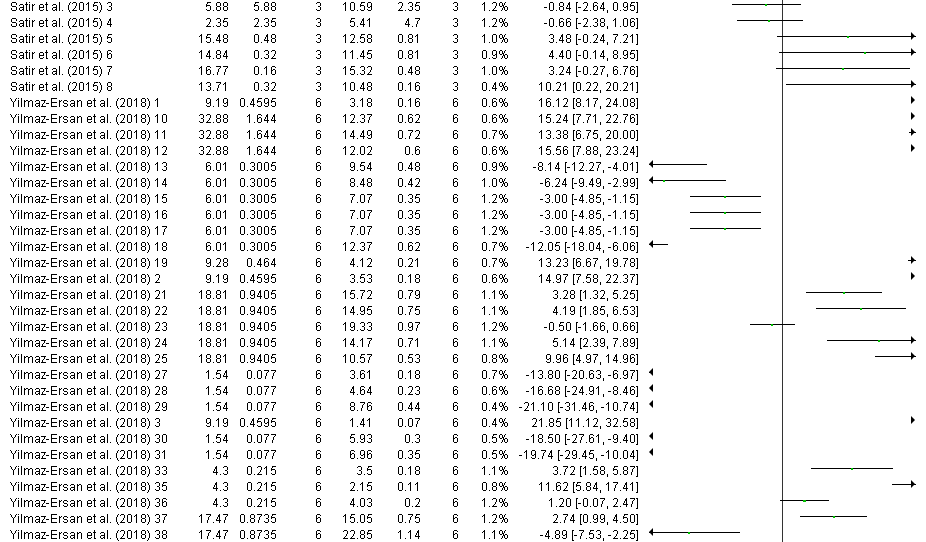

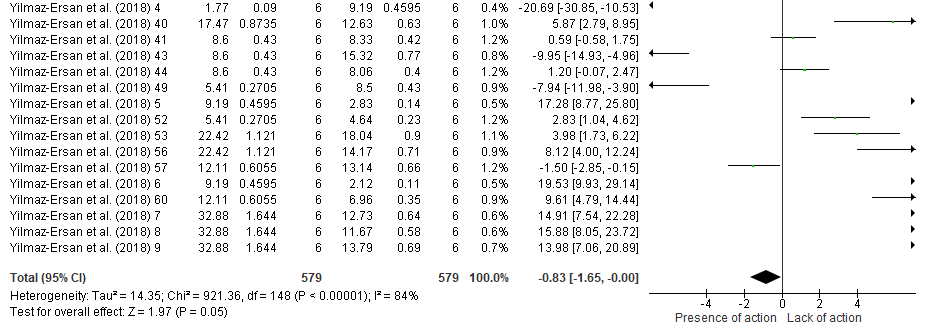


**Supplementary Figure S2.** Forest plot of the standardized mean difference (SMD) and 95% confidence intervals for studies concerning the presence or lack of antioxidant effect of kefir bioactive compounds compared to control treatments. Each line contains the point outcomes and confidence intervals. Green boxes represent the relative weight of each study. Combined effects estimates are represented by a diamond symbol at the bottom of each category.


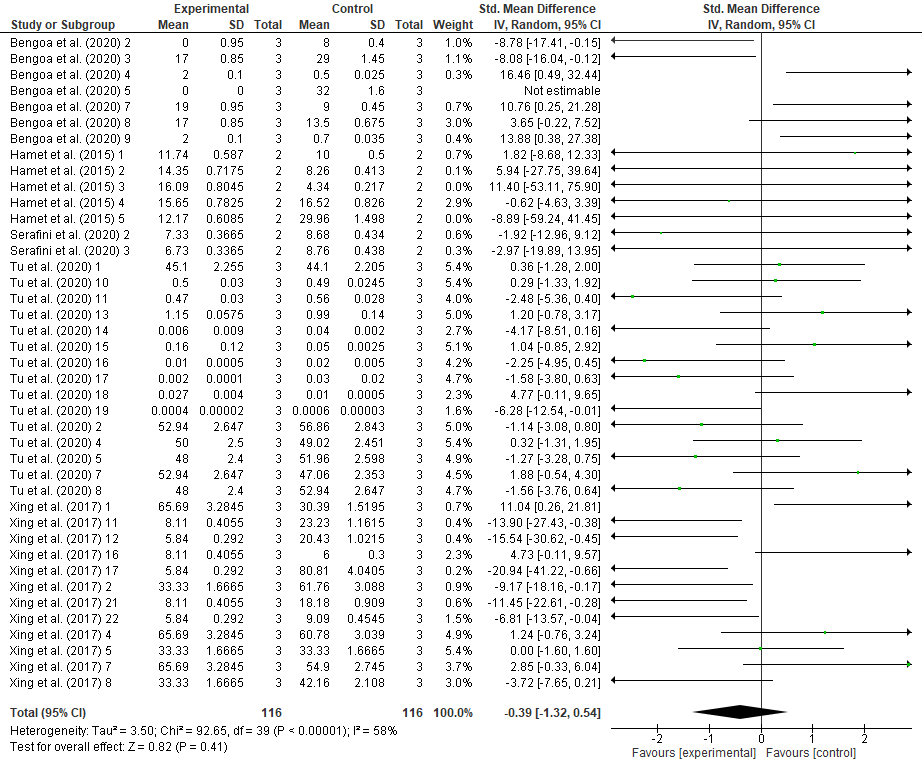


**Supplementary Figure S3.** Forest plot of the standardized mean difference (SMD) and 95% confidence intervals for studies concerning the presence or lack of gut microbiota modulation effect of kefir bioactive compounds compared to control treatments. Each line contains the point outcomes and confidence intervals. Green boxes represent the relative weight of each study. Combined effects estimates are represented by a diamond symbol at the bottom of each category.


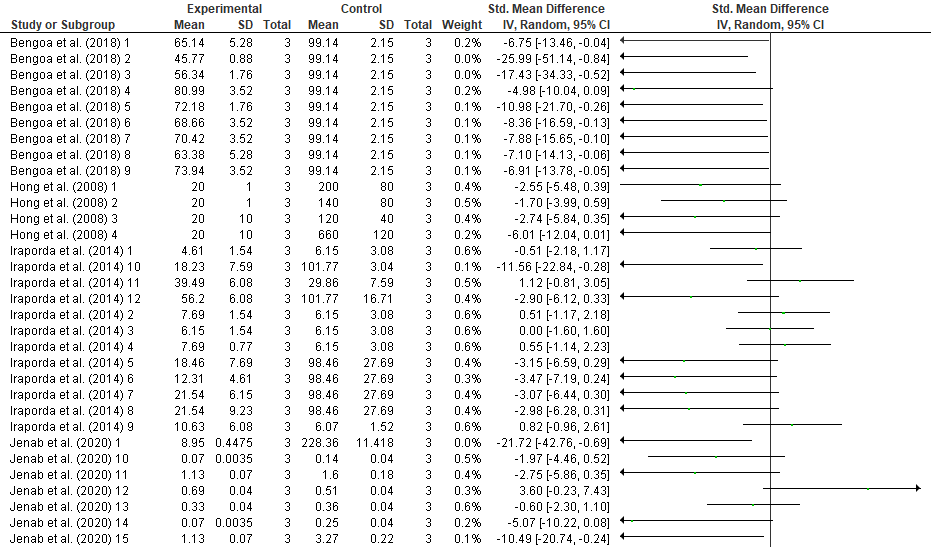

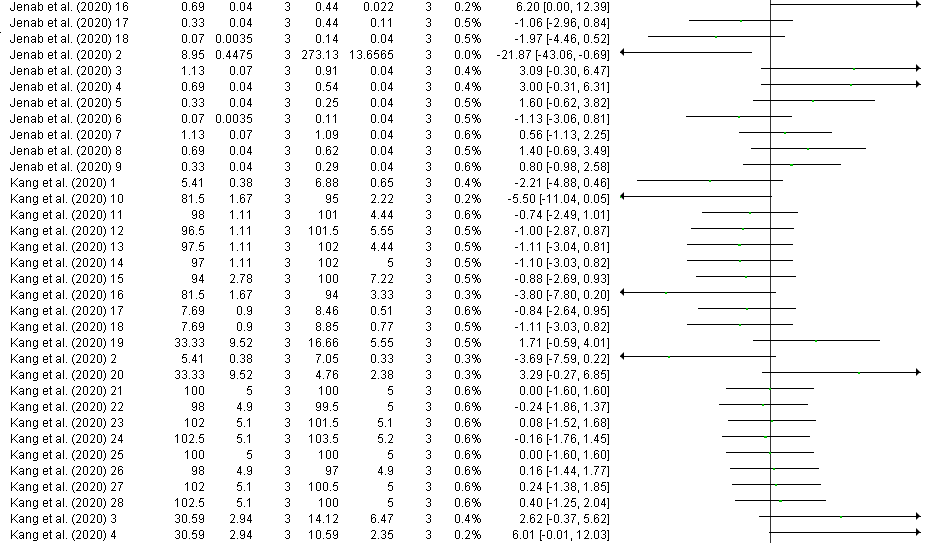

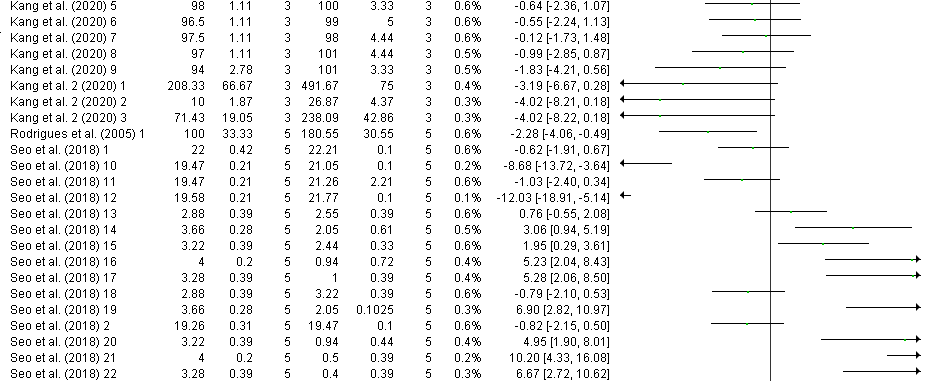


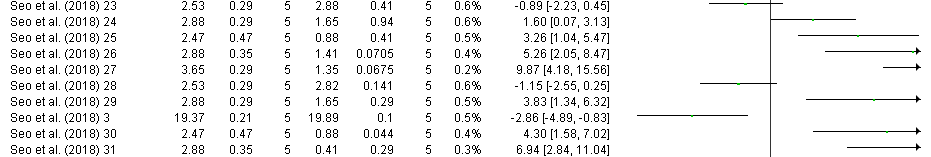


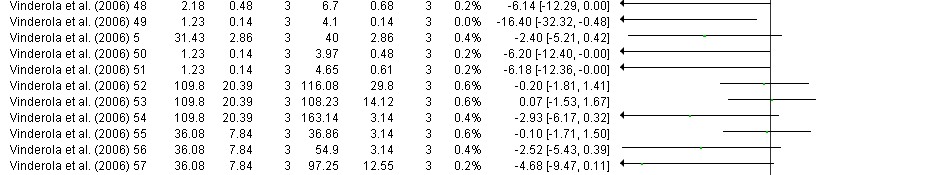

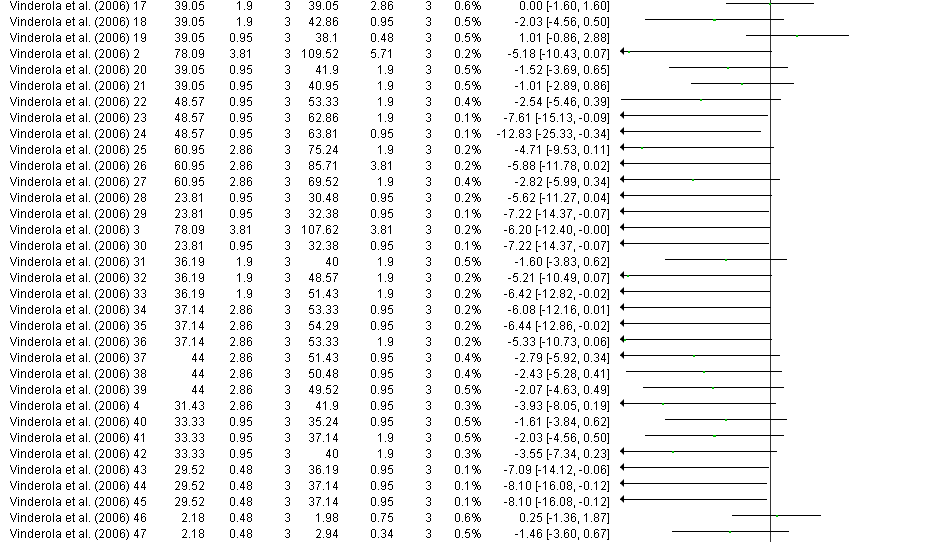

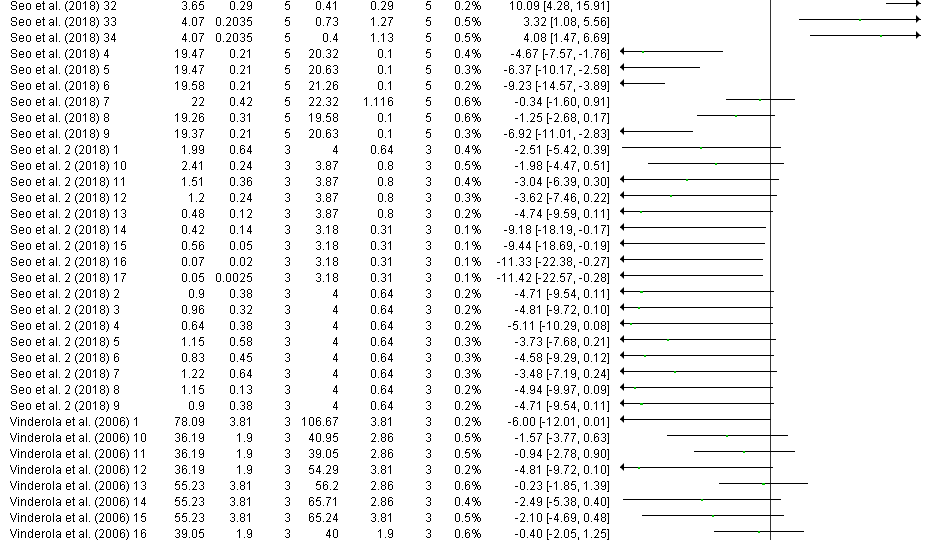


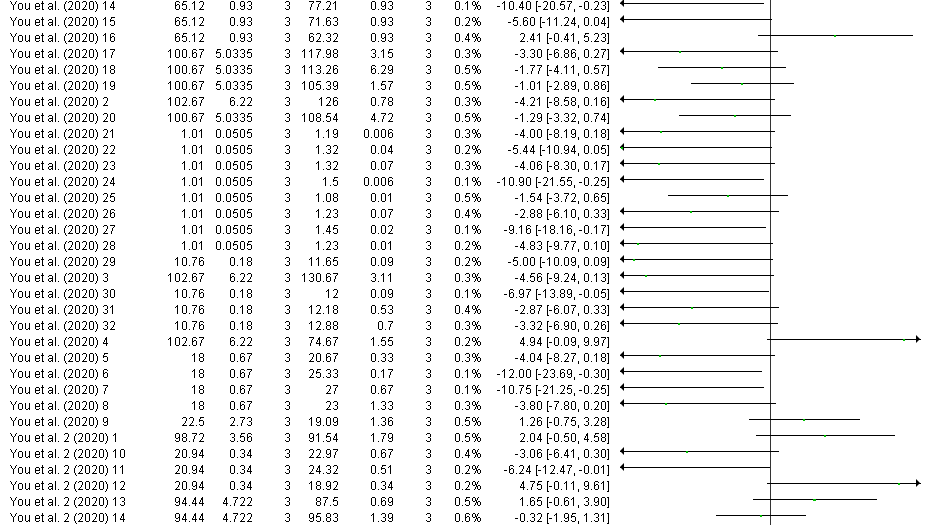

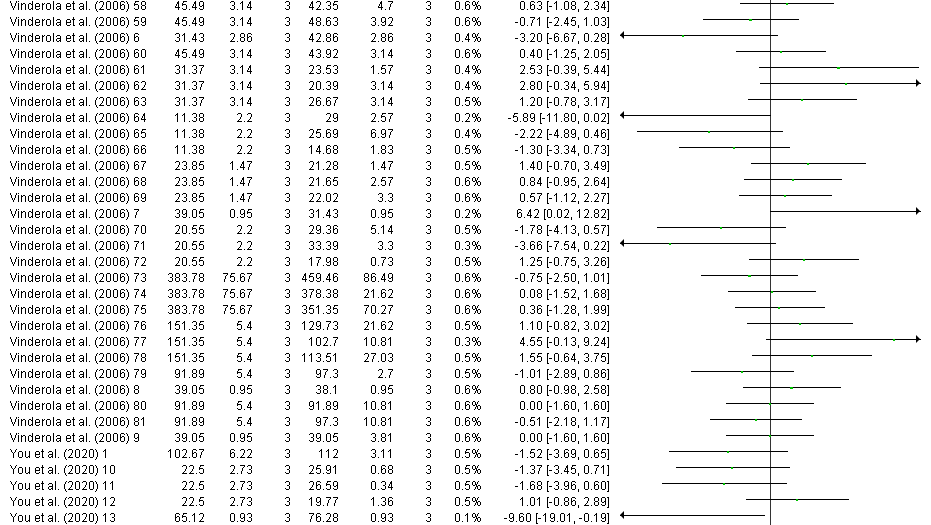


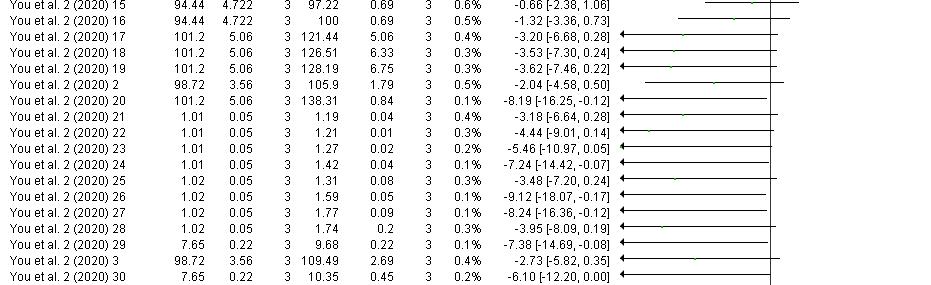


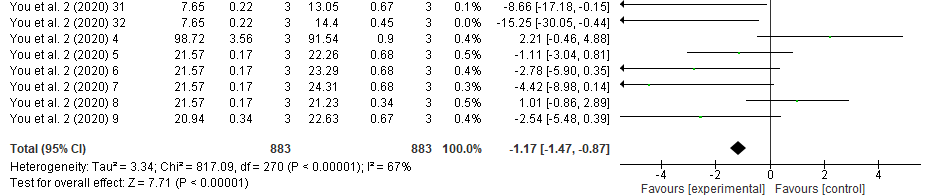


**Supplementary Figure S4.** Forest plot of the standardized mean difference (SMD) and 95% confidence intervals for studies concerning the presence or lack of immune modulation effect of kefir bioactive compounds compared to control treatments. Each line contains the point outcomes and confidence intervals. Green boxes represent the relative weight of each study. Combined effects estimates are represented by a diamond symbol at the bottom of each category.


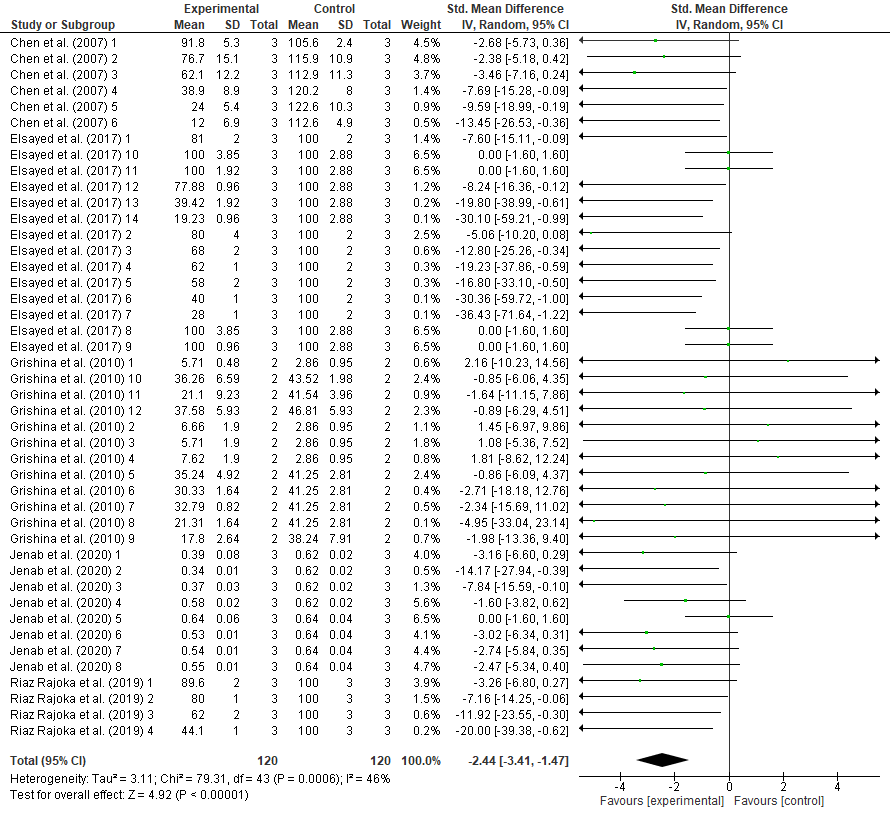


**Supplementary Figure S5.** Forest plot of the standardized mean difference (SMD) and 95% confidence intervals for studies concerning the presence or lack of anticarcer effect of kefir bioactive compounds compared to control treatments. Each line contains the point outcomes and confidence intervals. Green boxes represent the relative weight of each study. Combined effects estimates are represented by a diamond symbol at the bottom of each category.
